# Longitudinal Study of Shiga Toxin-Producing *Escherichia coli* and *Campylobacter jejuni* on Finnish Dairy Farms and in Raw Milk

**DOI:** 10.1128/AEM.02910-18

**Published:** 2019-03-22

**Authors:** Anniina Jaakkonen, Hanna Castro, Saija Hallanvuo, Jukka Ranta, Mirko Rossi, Joana Isidro, Miia Lindström, Marjaana Hakkinen

**Affiliations:** a Microbiology Research Unit, Finnish Food Safety Authority Evira, Helsinki, Finland; b Department of Food Hygiene and Environmental Health, Faculty of Veterinary Medicine, University of Helsinki, Helsinki, Finland; c Risk Assessment Research Unit, Finnish Food Safety Authority Evira, Helsinki, Finland; d BIOCONTAM Unit, European Food Safety Authority, Parma, Italy; e National Reference Laboratory for Gastrointestinal Infections, Department of Infectious Diseases, National Institute of Health Doutor Ricardo Jorge, Lisbon, Portugal; f Innovation and Technology Unit, Department of Human Genetics, National Institute of Health Doutor Ricardo Jorge, Lisbon, Portugal; Centers for Disease Control and Prevention

**Keywords:** *Campylobacter jejuni*, *Escherichia coli*, STEC, cattle, genomics, milk

## Abstract

Shiga toxin-producing Escherichia coli (STEC) and Campylobacter jejuni are notable health hazards associated with the consumption of raw milk. These bacteria may colonize the intestines of asymptomatic cattle and enter bulk tank milk via fecal contamination during milking. We studied the frequency of STEC O157:H7 and C. jejuni contamination in tank milk (*n* = 785) and the in-line milk filters of milking machines (*n* = 631) versus the frequency of isolation from cattle feces (*n* = 257) on three Finnish dairy farms for 1 year. Despite simultaneous isolation of STEC O157:H7 (17%) or C. jejuni (53%) from cattle, these bacteria were rarely isolated from milk filters (2% or <1%, respectively) and milk (0%). As revealed by phylogenomics, one STEC O157:H7 strain at a time was detected on each farm and persisted for ≤12 months despite rigorous hygienic measures. C. jejuni strains of a generalist sequence type (ST-883 and ST-1080) persisted in the herds for ≥11 months, and several other C. jejuni types were detected sporadically. The *stx* gene carried by STEC was detected more frequently from milk filters (37%) than from milk (7%), suggesting that milk filters are more suitable sampling targets for monitoring than milk. A questionnaire of on-farm practices suggested lower *stx* contamination of milk when major cleansing in the barn, culling, or pasturing of dairy cows was applied, while a higher average outdoor temperature was associated with higher *stx* contamination. Because pathogen contamination occurred despite good hygiene and because pathogen detection from milk and milk filters proved challenging, we recommend heat treatment for raw milk before consumption.

**IMPORTANCE** The increased popularity of raw milk consumption has created demand for relaxing legislation, despite the risk of contamination by pathogenic bacteria, notably STEC and C. jejuni. However, the epidemiology of these milk-borne pathogens on the herd level is still poorly understood, and data are lacking on the frequency of milk contamination on farms with cattle shedding these bacteria in their feces. This study suggests (i) that STEC contamination in milk can be reduced, but not prevented, by on-farm hygienic measures while fecal shedding is observable, (ii) that milk filters are more suitable sampling targets for monitoring than milk although pathogen detection from both sample matrices may be challenging, and (iii) that STEC and C. jejuni genotypes may persist in cattle herds for several months. The results can be utilized in developing and targeting pathogen monitoring and risk management on the farm level and contributed to the revision of Finnish legislation in 2017.

## Abstract (precis)

The increased popularity of raw milk consumption has created demand for relaxing legislation, despite the risk of contamination by pathogenic bacteria, notably STEC and C. jejuni. However, the epidemiology of these milk-borne pathogens on the herd level is still poorly understood, and data are lacking on the frequency of milk contamination on farms with cattle shedding these bacteria in their feces. This study suggests (i) that STEC contamination in milk can be reduced, but not prevented, by on-farm hygienic measures while fecal shedding is observable, (ii) that milk filters are more suitable sampling targets for monitoring than milk although pathogen detection from both sample matrices may be challenging, and (iii) that STEC and C. jejuni genotypes may persist in cattle herds for several months. The results can be utilized in developing and targeting pathogen monitoring and risk management on the farm level and contributed to the revision of Finnish legislation in 2017.

## INTRODUCTION

Raw cow milk consumption has become more popular in recent years, especially among urban consumers, creating demand for relaxing the legislation restricting raw milk sales (1–3). Releasing the sales of raw milk challenges food safety management through the milk production chain, which has relied on pasteurization to eliminate pathogenic bacteria. Related to the consumption of unpasteurized milk, Shiga toxin-producing Escherichia coli (STEC) and thermophilic *Campylobacter* spp., predominantly Campylobacter jejuni, have been regarded as the most notable health hazards, along with *Salmonella* spp. (1, 2). These pathogens have caused several milk-borne disease outbreaks and appear to be prevalent in cattle (3). Both STEC and C. jejuni are carried by asymptomatic cattle in their intestines and shed intermittently into the feces (4, 5). These pathogens may therefore enter milk via fecal contamination during milking. As suggested, pathogen contamination of milk may be reduced by good farming practices and milking hygiene but not prevented completely (6).

To evaluate the risk of STEC and *Campylobacter* infection in relation to the consumption of raw milk, previous studies have investigated the prevalence of STEC and thermophilic *Campylobacter* spp. in bulk tank milk. In addition, in-line milk filters of milking machines have been studied as indicators for milk contamination by STEC and *Campylobacter* spp. because all the milk entering the tanks passes through them. A recent meta-analysis estimated mean prevalences of 1.54% for *Campylobacter* spp. in bulk tank milk and 1.75% in milk filters, considering previously published data from North America, Europe, and New Zealand (7). However, reported prevalences varied notably between various studies and regions. According to the global review by Farrokh et al. (4), isolation rates of 0 to 2% have typically been reported for STEC from raw milk. However, high prevalence of *stx* genes, as indicators for STEC contamination, has been reported from both bulk tank milk (15.2%) and milk filters (51.0%), highlighting the higher sensitivity of molecular detection than of culture methods (8).

Despite numerous prevalence studies of STEC and thermophilic *Campylobacter* spp. in milk and milk filters, few studies have concentrated on the herd-level epidemiology of these milk-borne pathogens (9–12). Moreover, limited longitudinal data have been published on milk contamination on farms with cattle shedding these pathogens in their feces. As pathogen contamination of milk is expected to occur sporadically, frequent sampling is required to detect contamination events and draw conclusions on their frequency. In addition, detection of dilute pathogen contamination in large volumes of bulk tank milk poses a challenge, which likely necessitates several subsamples (13). Previous herd-level studies have analyzed a maximum of two milk subsamples once per month (9–12).

To avoid the aforementioned pitfalls in our present study, three dairy farms with previously detected carriage of STEC O157:H7 and C. jejuni were sampled for a period of 1 year: weekly for in-line milk filters and five subsamples of bulk tank milk and monthly for feces and the farm environment. First, we aimed to determine the frequency of milk contamination by STEC O157:H7 and C. jejuni and associate the occurrence of milk contamination with fecal shedding of these pathogens. Second, we compared bulk tank milk and milk filters as sampling targets for monitoring these pathogens. Third, we investigated the genomic variation of STEC O157:H7 and C. jejuni isolates to recognize on-farm persistence and contamination routes. Finally, on-farm practices were investigated with the aim of recognizing risk factors for milk contamination.

## RESULTS

### STEC O157:H7 was isolated from cattle feces on all three farms and infrequently from the barn environment.

Fecal samples from both milking cows and juvenile cattle were collected on three dairy farms between January 2014 and June 2015. Fecal carriage of STEC O157:H7 had been detected on the farms years or months before the commencement of our study, and since the initial detection the farms had implemented intensive hygienic measures to reduce the pressure for fecal-oral transmissions.

STEC O157:H7 was isolated from cattle feces (17%) on each farm (1 to 40%) during the study (Tables 1 to 4). The isolation rate was higher on farms 2 and 3 with more recently observed carriage. On farm 1, STEC O157:H7 was isolated only from juvenile cattle in September 2014. On farm 2, the isolation rate of STEC O157:H7 from cattle feces gradually decreased from the commencement of the study, from 92% to 0% between March and July, and no positives were detected after six months (September 2014). On farm 3, STEC O157:H7 was detected in 20 to 50% of the fecal samples from juvenile cattle at the commencement of the study (February to May) and in 100% of the fecal samples from milking cows two months later, in July 2014. STEC O157:H7 was infrequently isolated from drinking troughs: 0 to 6% of the samples tested positive on each farm (Table 1). Drinking water was sampled only on farm 1, and STEC O157 was not detected.

**TABLE 1:** Occurrence of Shiga toxin-producing Escherichia coli (STEC) and Campylobacter jejuni in feces, drinking troughs, milk, and milk filter samples on three dairy farms during 1 year

Analysis method and sample source	No. of positive samples/no. of analyzed samples (%)^*a*^			
	Farm 1	Farm 2	Farm 3	Total
STEC O157:H7 culture				
Feces, in total	1/87 (1)	34/85 (40)	9/85 (11)	44/257 (17)
Feces, milking cows	0/35 (0)	22/69 (32)	6/60 (10)	28/164 (17)
Feces, juvenile cattle	1/52 (2)	12/16 (75)	3/25 (12)	16/93 (17)
Drinking troughs	0/85 (0)	4/65 (6)	3/49 (6)	7/199 (4)
Milk	0/260 (0)	0/260 (0)	0/269 (0)	0/789 (0)
Milk filters	0/141 (0)	8/318 (3)	4/173 (2)	12/632 (2)
STEC non-O157 culture				
Milk	0/260 (0)	0/260 (0)	2/269 (1)^*c*^	2/789 (<1)
Milk filters	0/141 (0)	1/318 (<1)^*b*^	5/173 (3)^*d*^	6/632 (1)
PCR screening for *stx* in STEC				
Milk	9/260 (3)	25/260 (10)	18/269 (7)	52/789 (7)
Milk filters	21/141 (15)	142/317 (45)	70/173 (40)	233/631 (37)
PCR screening for *stx* and *eae* in STEC				
Milk	2/260 (1)	15/260 (6)	15/269 (6)	32/789 (4)
Milk filters	6/141 (4)	108/317 (34)	64/173 (37)	178/631 (28)
C. jejuni culture				
Feces, in total	14/87 (16)	48/85 (56)	74/85 (87)	136/257 (53)
Feces, milking cows	11/35 (31)	46/69 (67)	58/60 (97)	115/164 (70)
Feces, juvenile cattle	3/52 (6)	2/16 (13)	16/25 (64)	21/93 (23)
Drinking troughs	0/85 (0)	1/65 (2)	9/49 (18)	10/199 (5)
Milk	0/260 (0)	0/260 (0)	0/265 (0)	0/785 (0)
Milk filters	0/140 (0)	0/318 (0)	1/173 (1)	1/631 (<1)

a For milk data, the number of positive subsamples was used.

b Serotype O182:H25.

c Serotype O121:H19.

d Serotype O26:H11.

**TABLE 2:** Occurrence of Shiga toxin-producing Escherichia coli (STEC) and Campylobacter jejuni in fecal samples of farm 1

Organism and sample source	No. of positive fecal samples per sampling (%)										
	February 2014	March 2014	April 2014	May 2014	June 2014	July 2014	August 2014	September 2014	October 2014	December 2014	February 2015
STEC O157:H7											
Cows	0	0	0	0	0	0	0	0	0	0	0
Juveniles	0	0	0	0	0	0	0	1 (17)	0	0	0
C. jejuni											
Cows	3 (60)	1 (25)	0	3 (100)	0	0	0	0	2 (67)	1 (33)	0
Juveniles	0	2 (40)	0	0	0	1 (17)	0	0	0	0	0

**TABLE 3:** Occurrence of Shiga toxin-producing Escherichia coli (STEC) and Campylobacter jejuni in fecal samples of farm 2

Organism and sample source	No. of positive fecal samples per sampling (%)^*a*^										
	March 2014	April 2014	May 2014	June 2014	July 2014	August 2014	September 2014	November 2014	January 2015	March 2015	May 2015
STEC O157:H7											
Cows	7 (88)	6 (86)	4 (57)	1 (17)	0	1 (17)	3 (60)	0	0	0	0
Juveniles	5 (100)	2 (50)	3 (100)	1 (100)	NA	0	1 (50)	NA	NA	NA	NA
C. jejuni											
Cows	5 (63)	6 (86)	7 (100)	5 (83)	7 (100)	6 (100)	3 (60)	3 (50)	0	0	4 (80)
Juveniles	1 (20)	0	0	0	NA	0	1 (50)	NA	NA	NA	NA

a NA, not analyzed.

**TABLE 4:** Occurrence of Shiga toxin-producing Escherichia coli (STEC) and Campylobacter jejuni in fecal samples of farm 3

Organism and sample source	No. of positive fecal samples per sampling (%)^*a*^										
	February 2014	May 2014	June 2014	July 2014	August 2014	September 2014	October 2014	November 2014	January 2015	March 2015	May 2015
STEC O157:H7											
Cows	0	0	0	6 (100)	0	0	0	0	0	0	0
Juveniles	1 (20)	2 (50)	0	0	0	0	0	0	NA	NA	NA
STEC O26:H11											
Cows	NA	NA	NA	NA	NA	3 (50)	NA	NA	NA	0	1 (17)
Juveniles	NA	NA	NA	NA	NA	1 (50)	2 (67)^*b*^	1 (14)^*b*^	NA	NA	NA
C. jejuni											
Cows	1 (100)	5 (83)	5 (100)	6 (100)	6 (100)	5 (83)	6 (100)	6 (100)	6 (100)	6 (100)	6 (100)
Juveniles	4 (80)	4 (100)	0	0	0	2 (67)	1 (33)	5 (71)	NA	NA	NA

a NA, not analyzed.

b Nonspecific detection with O157 IMS.

### STEC non-O157 serogroups were isolated from cattle feces on farms 2 and 3.

In addition to STEC O157, the feces were cultured for other STEC serogroups if these serogroups (O157:H7, O26, O103, O145, O111, O121, and O45) were first detected in PCR screening from the milk or milk filters, as was the case on farms 2 and 3. The farms were monitored for other STEC serogroups to evaluate the influence of these serogroups on the PCR results as PCR cannot distinguish whether *stx* signals arise from STEC O157. Isolates of other serogroups were also occasionally recovered in conjunction with the attempted isolation of O157.

On farm 2, STEC O15:H16 was sporadically isolated from the feces of milking cows in May 2015. No additional serogroups were isolated, albeit the cattle were examined by culture for STEC O103 in March and April 2014 and for O121, O145, O26, and O45 in May 2015. On farm 3, STEC O26:H11 was isolated from cattle feces during four samplings between August 2014 and June 2015 and once from the drinking troughs (Table 4). STEC O84:H2 was sporadically isolated from milking cows in October 2014. None of the STEC serogroups O103, O121, O45, and O145 were isolated from cattle feces in May 2015.

### STEC was seldom isolated from milk or milk filters on farms 2 and 3.

**FIG 1:**
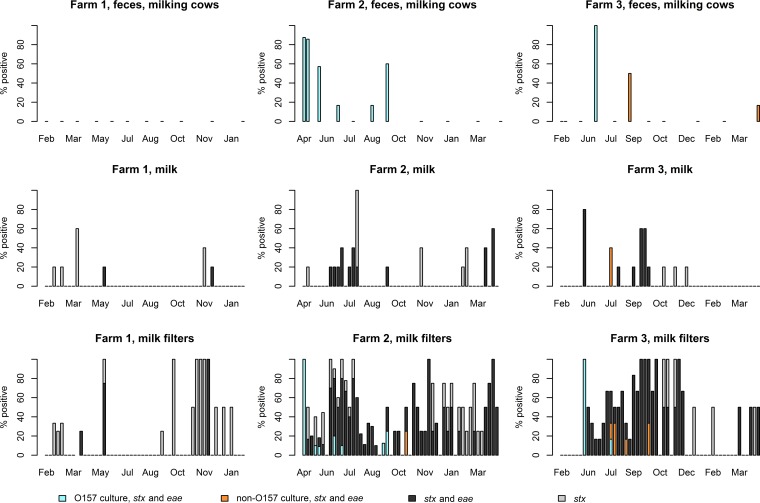
Detection rates of STEC from the feces of milking cows, milk subsamples, and milk filters in different samplings on three dairy farms between January 2014 and June 2015. An STEC non-O157 isolate that harbored only *stx* was additionally recovered from feces on farm 2 in May 2015.

STEC O157:H7 was seldom isolated from milk filters: 2% of the samples tested positive (0 to 3% on each farm) (Table 1 and Fig. 1). Culture-positive milk filters were observed only on farms 2 and 3 and only simultaneously with fecal isolation from milking cows. No STEC O157 isolates were obtained from milk.

In addition to O157, other STEC serogroups were also studied using a culture method during each sampling, and other STEC serogroups were isolated from milk (<1%) and milk filters (1%) during the study (Table 1 and Fig. 1). On farm 2, STEC O182:H25 was isolated once from milk filters in October 2014. On farm 3, STEC O121:H19 was isolated once from milk in July 2014, and STEC O26:H11 was isolated from five milk filter samples in July to October 2014. Altogether, STEC of any serogroup was isolated from milk filters in 13 (8%) samplings, and only in four samplings were isolates recovered from more than one milk filter sample.

### Milk filter samples tested PCR positive for *stx* more frequently than milk.

All milk and milk filter samples were screened for the virulence genes *stx* and *eae* by PCR. *stx* was infrequently detected from milk: 7% of the subsamples tested positive (3 to 10% on each farm) (Table 1 and Fig. 1). Altogether, *stx* was detected from milk in 30 (19%) samplings (12 to 27% on each farm). Of these 30 samplings, only one subsample tested positive in the majority (57%) of samplings, and three or more subsamples were positive in 20% of the samplings. In 90% of the 30 samplings, the milk filter samples also tested *stx* positive.

Milk filter samples tested positive for *stx* (37%) more frequently than milk subsamples (7%) (Table 1 and Fig. 1). *stx* was detected from milk filters in 91 (58%) samplings (29 to 86% on each farm). In 30% of these 91 samplings, *stx* was also detected in the milk subsamples. Overall, *stx* prevalence appeared 5.6-fold (4.3- to 6.0-fold on each farm) higher in milk filter samples than in milk subsamples, with an odds ratio of 8.3 (95% confidence interval, 6.0 to 11.5) for higher prevalence in milk filters. Samplings with *stx*-positive milk filter samples occurred 3.1-fold more often than samplings with *stx*-positive milk samples, with an odds ratio of 5.9 (95% confidence interval: 3.6 to 9.9) for higher occurrence in milk filters.

### Detection rate of *stx*, *eae*, and serogroup-specific genes by PCR varied farm to farm.

*stx* and *eae* were detected from milk and milk filters also when no STEC could be isolated from cattle feces. However, farm-to-farm variation was observed: 3-fold more *stx*-positive milk (7 to 10%) and milk filters (40 to 45%) were detected on farms 2 and 3 than on farm 1 (3% and 15%, respectively) where STEC was not isolated from fecal samples of milking cows during the study (Table 1 and Fig. 1). On farm 1, only 29% of the *stx*-positive milk filters also tested positive for *eae*, whereas the majority of the *stx*-positive milk filters tested *eae* positive on farms 2 (76%) and 3 (91%).

Milk and milk filters positive for *stx* and *eae* were also PCR screened for seven serogroups associated with the highest health risk for consumers (14). Serogroups O157:H7, O103/O145, O26, O45, and O121 were detected with PCR from milk or milk filters on farms 2 and 3. No serogroup O111 was detected. Few of the examined filters, i.e., 17 (17%) and 1 (2%) on farms 2 and 3, respectively, tested positive by PCR for O157:H7. STEC O157:H7 was not detected by PCR from two milk filters, despite isolates being obtained, indicating insensitivity of the PCR method for O157:H7. On farm 2, fewer (0 to 15%) examined milk filters (*n* = 39) tested positive by PCR for serogroups other than O157:H7. On farm 3, a higher proportion (31 to 48%) of the examined milk filters (*n* = 29) tested positive by PCR for O45, O26, and O121 than for O157:H7 and the other serogroups (0 to 7%), congruent with the isolation of O26 and O121 from the farm.

### STEC isolates shared similar genetic features within a serogroup.

STEC isolates were studied for serotype and the main virulence genes, and one or two isolates were subtyped per each positive sample (feces or other sample materials, respectively) by pulsed-field gel electrophoresis (PFGE). All STEC O157:H7 isolates from the three farms harbored virulence genes *stx*_1a_, *stx*_2c_, *eae*, and *hlyA*, represented clade 7 of Manning et al. (15), and were highly similar with each other in PFGE (similarity, ≥95%; maximum difference of four bands from the predominant type). In addition, STEC O26:H11 isolates from farm 3 represented highly similar pulsotypes with each other (similarity, ≥95%; maximum difference of two bands from the predominant type) and harbored genes *stx*_1a_, *eae*, and *hlyA*. Other STEC isolates were sporadic findings from farm 2 (O15:H16 harboring *stx*_2g_ and *estIa*; O182:H25 harboring *stx*_1a_, *eae*, and *hlyA*) and farm 3 (O84:H2 harboring *stx*_2c_, *eae*, and *hlyA*; O121:H19 harboring *stx*_2a_, *eae*, and *hlyA*) (see Table S1 in the supplemental material).

### Phylogenomics of STEC O157:H7 revealed persistence of one or two genotypes on each farm.

To conclude whether one or more STEC O157:H7 genotypes persisted on the farms, phylogenomic analysis was performed for a selection of farm isolates collected both before (1 to 5 isolates per farm) and during (2 to 16 isolates per farm) the study period (Table S1). Thus, the isolate selection also considered the first detection of the pathogen on the farm years or months before our study and represented all positive samplings. First, whole-genome multilocus sequence typing (wgMLST) was performed to investigate the similarity of 32 farm isolates among 482 globally collected isolates from the INNUENDO database (16), all representing sequence type 11 (ST-11) in seven-locus multilocus sequence typing (MLST) (Data Set S1). In the resulting minimum spanning tree (Fig. S1), the farm isolates grouped with other Finnish isolates (from cattle, farm environments, or human clinical samples) in a separate branch from foreign isolates and appeared to be within relatively close pairwise distances (PWDs). In the allelic profile size of 2,353, the maximum PWD was 23 (1.0%) among the farm isolates, while the maximum PWD within the whole data set was 633 (27%). The closest foreign isolate, collected in the United Kingdom in 2016, differed from the closest farm isolate, with a PWD of 30 (1.3%), and was selected as an outgroup for further analyses.

**FIG 2:**
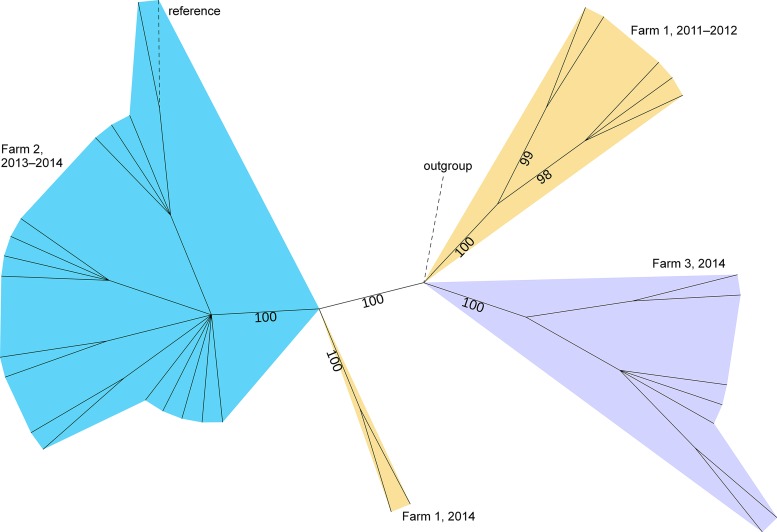
Maximum likelihood tree based on genome-wide SNPs of 32 STEC O157:H7 isolates, collected on three dairy farms from October 2011 through November 2014. SNPs were called against the in-group reference (Ec_Farm2_2014-03_C1). An outgroup strain (ESC_FA0769AA), collected in the United Kingdom in 2016, was included in the analysis. The tree is unrooted, and UFBoot support values ≥95% are shown. Branch lengths are ignored, and only branches with support values of ≥80 are shown. Lineages are colored by farm. Branches of the in-group reference and outgroup are denoted by dashed lines. The tree was visualized using iTOL (75).

Because of the high similarity between the farm isolates, their phylogeny was investigated with higher resolution by mapping the sequencing reads to a draft, in-group reference genome to determine genome-wide single nucleotide polymorphisms (SNPs). The resulting maximum likelihood tree, which considered both SNPs and invariant sites, revealed four lineages with high confidence, i.e., UFBoot bootstrapping support of 100% (Fig. 2). All isolates from farm 2, collected during a 12-month period (October 2013 to September 2014), clustered into one lineage. Likewise, one lineage grouped the isolates from farm 3, collected during an 11-month period (January to November 2014). The isolates of farm 1 divided into two lineages: one lineage for the isolates collected in October 2011 to January 2012 and one lineage for the isolates collected more than 2.5 years later, in September 2014. Thus, a maximum 12 months of on-farm persistence was detected for STEC O157:H7 strains in our study, within the limits of sampling periods. Similar results were obtained both by including and excluding the outgroup strain from the analysis. To further date back the closest ancestor of farm 1 lineages, temporal signal and validity of the molecular clock assumption were investigated using root-to-tip analysis. No clear temporal signal was detected in this analysis (*R*^2^ =1.6 × 10^−3^), and, thus, no evolutionary dating was performed.

### No C. jejuni could be isolated from milk although it appeared prevalent in cattle.

C. jejuni was isolated only from one milk filter sample and not from milk in our study (Table 1). Nevertheless, C. jejuni was repeatedly isolated from cattle feces: 53% of the samples tested positive (16 to 87% on each farm). As with the fecal samples, swab samples from the drinking troughs showed farm-to-farm variation (0 to 18%). Drinking water was sampled only on farm 1, and no C. jejuni could be isolated from these samples.

### C. jejuni was isolated only in summer on farm 2, and only sporadic genotypes were detected.

One or two C. jejuni isolates from each positive sample (feces or other sample materials, respectively) were subtyped by PFGE, and a representative isolate of each pulsotype was subjected to whole-genome sequencing, followed by MLST (Table S2 and Fig. S2). Persistent pulsotypes detected in more than four samplings were further investigated using wgMLST.

On farm 2, the isolation rate of C. jejuni from the fecal samples of milking cows remained high (≥83%) during the warm months (May to August) and gradually decreased to 0% in winter (Table 3). Only once was C. jejuni recovered from the drinking troughs. The isolates represented 15 pulsotypes, which appeared in one to four temporally related samplings, and no persistent pulsotypes were recognized. Furthermore, the pulsotypes represented eleven STs (11, 45, 48, 267, 677, 692, 1701, 1938, 4080, 5559, and 7435). Six farm isolates of ST-45 were further subjected to wgMLST with 436 ST-45 isolates from the INNUENDO database (Data Set S2 and Fig. S3) (17). In the allelic profile size of 654 loci, dissimilarity was observed between the isolates that were collected from farm 2 in spring and fall (PWD, 44.6%) and between isolates from the three farms (PWD, ≥3.5%), supporting sporadic findings.

### Both sporadic and persistent C. jejuni genotypes were detected on farms 1 and 3.

On farm 1, the isolation rate of C. jejuni from cattle feces varied over time, with no recognizable trend (Table 2). The C. jejuni isolates represented three pulsotypes that were detected sporadically by MLST and belonged to ST-45 or ST-1080 (Table S2 and Fig. S2). One pulsotype that represented ST-1080 appeared persistently in five samplings throughout the year 2014. Three ST-1080 isolates of farm 1, collected during an 11-month period (February to December 2014), were subjected to wgMLST and compared with four ST-1080 isolates available from the INNUENDO database (17), all originating from Finland (Data Set S3 and Fig. S4). In the allelic profile size of 874 loci, the isolates from farm 1 appeared within a PWD of 2 (0.2%) from each other and a PWD of 22 to 28 (2.5 to 3.2%) from the other Finnish ST-1080 isolates. Thus, the ST-1080 isolates of farm 1 appeared similar in wgMLST, supporting PFGE results on the persistence of a single strain.

On farm 3, a high proportion (≥83%) of the fecal samples from milking cows tested positive for C. jejuni in every sampling (Table 4). In addition, C. jejuni could be isolated from drinking troughs (18%) in four samplings, indicating defects in the hygiene and pressure for fecal-oral transmissions. Eighteen pulsotypes were recognized among the C. jejuni isolates, representing eleven STs (45, 267, 538, 583, 883, 925, 991, 4080, 5559, 9407, and 9408). A majority of the C. jejuni pulsotypes appeared sporadically in one or two samplings. One pulsotype (ST-4080 in MLST) was detected in four samplings throughout the year. Furthermore, one pulsotype (ST-883 in MLST) occurred persistently in each sampling after initial detection in July 2014. In addition to detection in feces, ST-883 was recovered twice from drinking troughs and once from milk filters.

Five ST-883 isolates from farm 3 were further subjected to wgMLST and compared with 66 ST-883 isolates of global origin from the INNUENDO database (Data Set S4 and Fig. S5). Farm 3 isolates had been recovered during an 11-month period (July 2014 to May 2015) and appeared within a PWD of 1 (0.1%) from each other in the allelic profile size of 801 loci. Overall, the maximum PWD within all 71 isolates was 285 (36%) and the closest other isolates (from Austria) were within PWDs of 14 to 15 (1.7 to 1.9%) from the isolates of farm 3. Results obtained by wgMLST indicated a high similarity between the ST-883 isolates from farm 3, thus supporting PFGE results on persistence.

### On-farm practices and temperature affected *stx* contamination of milk.

To recognize risk factors for milk contamination, we investigated the effect of on-farm practices, meteorological factors, and hygiene indicators on *stx* contamination of milk using a logistic regression model with nine explanatory variables (Table 5). *stx* contamination of milk was reduced by three on-farm practices: removal of milking cows from the herd by culling (variable “Sale”), major cleansing in the barn (variable “Cleanse”), and pasturing of milking cows (variable “Pastured”). Average outdoor temperature (variable “Temp”) weakly increased milk contamination (95% credibility interval, 0.42 to 1.34). However, mild correlation (Pearson coefficient of 0.6) was observed between temperatures (Temp) and pasturing (Pastured) in the data exploration, which could have weakened the observed effects of these variables.

**TABLE 5:** Explanatory variables and their modeled effects on the contamination of bulk tank milk by the *stx* gene that indicates contamination by STEC bacteria^*a*^

Variable name	Description	Posterior probability *P*(β*_k_* > 0)	95% posterior credibility interval	Effect
Farm	Fixed effect with three levels			
Sale	Culling, i.e., removal of cows from the dairy herd	0.0002	−72.14, −2.58	Negative
Cleanse	Major cleansing in the barn	0.008	−5.59, −0.39	Negative
Feed	Abnormalities in feed	0.68	−0.77, 1.10	None
Pastured	Pasturing of milking cows	0.01	−2.14, −0.16	Negative
Maint	Maintenance and breaks of the milking equipment	0.07	−3.08, 0.27	None
Rain	Number of rainy days (≥1 mm) during 6 days preceding the sampling	0.52	−0.23, 0.24	None
Temp	Average outdoor temperature (°C) during 6 days preceding the sampling	1.00	0.42, 1.34	Positive
Bact	Total bacterial counts (1,000 CFU/ml)	0.79	−0.21, 0.30	None
Cell	Somatic cell counts (1,000/ml)	0.40	−0.41, 0.29	None

a Data consisted of weekly questionnaire answers on farm practices, meteorological observations, and laboratory results on milk quality on three dairy farms during 1 year.

No effect on *stx* contamination of milk was observed for five variables: abnormalities in feed (variable “Feed”), maintenance of the milking equipment (variable “Maint”), rainy days as an indicator of humidity (variable “Rain”), total bacterial counts (variable “Bact”), and somatic cell counts (variable “Cell”). However, maintenance of the milking equipment (Maint) possibly reduced milk contamination (95% credibility interval: −3.08 to 0.27) but exceeded the tolerance limit for uncertainty, and, thus, reliable conclusions cannot be drawn.

## DISCUSSION

We studied the occurrence of STEC O157:H7 and C. jejuni in cattle feces to evaluate the contamination pressure on milk on three farms with previously detected carriage of these pathogens. Since the first detection of STEC O157:H7 years or months before our study, the farms had followed national policies and implemented rigorous hygienic measures to reduce environmental contamination and, thereby, fecal-oral transmissions. Therefore, these measures could have reduced the fecal prevalence over months and contributed to the variation observed between the samplings and farms although the observed variation could also have been due to naturally intermittent fecal shedding. Previous reports have suggested the effect of on-farm practices on STEC but not on C. jejuni (18, 19). Fecal shedding of STEC O157:H7 and C. jejuni was at least intermittently observed on each farm during the study, introducing a contamination pressure on milk via feces.

STEC O157:H7 was isolated from milk filters only simultaneously with the fecal isolation from milking cows, and no isolates were recovered from milk. However, other STEC serogroups were additionally isolated from the farms and could have contributed to the rate of *stx* detection. The detection of *stx* and serogroup-specific genes in milk and milk filters reflected the on-farm rate of STEC isolation from the feces of milking cows and serogroups of the isolates, suggesting that PCR results from milk and milk filters were associated with fecal shedding. We observed lower detection rates for *stx* and *eae* and no serogroup-specific genes on farm 1, where no STEC was isolated from cow feces. Positive PCR results in the absence of culturable STEC suggest shedding of the pathogen at levels below the detection limit of culture methods, intermittent shedding that was not captured by monthly samplings, or the presence of free-floating *stx*-converting bacteriophages (20). Because *stx* signals may arise from free phage particles, *eae* signals may arise from enteropathogenic E. coli and other species, and serogroup-specific genes may be from nonpathogenic E. coli (14), PCR cannot confirm the presence of viable STEC isolates that harbor these genes. However, high prevalence of *stx* phages in the farm environment could pose a risk for new STEC pathotypes by transduction (20). Overall, any STEC should be considered a potential pathogen to humans as novel pathogroups may arise from the rearrangements of virulence features due to high genomic plasticity (21).

Detection of STEC O157:H7 in cattle feces decreased below the detection limit on all three farms during the study period. The isolates represented clade 7, which has been associated with less severe disease and with environmental fitness that likely facilitates O157:H7 colonization of bovines (15, 22). As revealed by phylogenomic analysis, only one STEC O157:H7 lineage was detected on each farm at a time, and the lineages persisted for up to 12 months after the first detection. On farm 1, the detection of two lineages, sampled more than 2.5 years apart, suggested reintroduction of STEC O157:H7 to the herd after months of negative detection, assuming that the analyzed isolates (*n* = 7) well represented the on-farm genomic variation. Similarly, Worley et al. (23) found only one or two genomic lineages of STEC O157:H7 within herds in California regardless of the isolation rate, and they observed on-farm persistence of a lineage for 11 months. In our study, origin and source of the presumably new farm 1 strain were not investigated, but the results of wgMLST indicated a domestic origin: the strain grouped with other Finnish isolates, which mainly sourced from cattle, farm environments, or human clinical samples. Transmission from drinking water, purchased cattle, or feed was excluded because STEC was not detected from the water samples; and all new animal material was raised, and silage was produced on farm. In general, STEC survives in the environment and effluents for long periods of time and can be transmitted to cattle via animal vectors such as wildlife and pests (24).

In contrast to STEC O157:H7, C. jejuni was repeatedly isolated from cattle feces on all three farms but sparsely detected in milk and milk filters. Despite hygienic measures, persistence of C. jejuni was observed on two farms for at least 11 months, and sporadic strains were detected on all three farms. As suggested previously (25), certain C. jejuni genotypes have adapted and circulate within farm environments but cannot outcompete other genotypes as several genotypes are observed simultaneously within a herd. Furthermore, generalist genotypes that infect multiple animal species occur in agricultural environments, whereas genotypes in wildlife show more host specificity. On farm 2, high occurrence of sporadic C. jejuni only in summer suggests that transmissions originated from the surrounding environment outside the frost season (November to February). Pasturing in a bird-rich area may have contributed to the short-term colonization of animals by C. jejuni although transmissions between cattle and wild birds are controversial (25–27). Persistence of two C. jejuni genotypes, namely, ST-1080 (no clonal complex [CC] designation by the time of writing) and ST-883 (ST-21 CC), was observed on farms 1 and 3, respectively. Both STs and ST-21 CC have previously been found from multiple hosts (https://pubmlst.org/campylobacter/) (28), indicating the generalist nature of the genotypes. These genotypes could supposedly be adapted to long-term colonization of the animals or sustain the colonization in the herd via survival in the environment better than the sporadic genotypes. Various survival abilities of C. jejuni lineages have been demonstrated in sources outside the host (29, 30), but more research is needed on reservoirs and vehicles in farm environments.

Furthermore, we investigated the frequency of milk contamination on farms positive for STEC O157:H7 and C. jejuni and compared the suitability of bulk tank milk and milk filters as sampling targets. Milk entering a tank is passed through the milk filters, which could therefore indicate contamination during milking and be used as an alternative sampling target to bulk tank milk (8, 13, 31). More frequent detection of STEC and C. jejuni from milk filters than milk suggests that pathogen contamination occurred during milking but often could not be detected from the five milk subsamples. Detection was probably hindered by the low concentrations of pathogenic bacteria in the tank milk. This is supported by the observation that in the majority of samplings only one out of five subsamples tested positive. Pathogen contamination occurred despite the milk appearing of good hygienic quality, based on total bacterial counts that were usually below 50,000 CFU/ml on all three farms. No correlation between total bacterial counts and *stx* contamination of milk was observed in the statistical analysis. Similarly, Ruusunen et al. (6) observed that total bacterial counts poorly indicated pathogen contamination. However, dissimilar results on pathogen contamination of milk could have been obtained on farms with poor milking hygiene (31). Of note, failure to detect small quantities of STEC and C. jejuni in milk could pose serious public health implications as only a few cells may cause infection, depending on strain characteristics and host susceptibility (21, 32). As one positive out of five subsamples (25 ml) corresponds to 0.0089 most probable number (MPN)/ml (95% confidence interval, 0.0012 to 0.066 MPN/ml) (33), one glass (200 ml) of milk could cause infection with the contamination levels observed in our study.

As the detection of STEC from milk filters suggested, fecal contamination occurred during milking. Nevertheless, C. jejuni was poorly detected from milk filters and milk, despite the majority of fecal samples from milking cows testing positive. This may indicate poor survival of C. jejuni in both milk and milk filters or an inability to detect the pathogen by standard culture methods due to sublethal cell damage (7, 29). Milk filter samples were analyzed 48 to 72 h after sampling and storage in buffered peptone water, which appeared insufficient for the detection of C. jejuni. As recently suggested by Artursson et al. (13), the isolation rate of C. jejuni from milk filters is highly affected by sampling regime and could be improved by the addition of Cary Blair transport medium. On the other hand, C. jejuni strains may differ in their abilities to survive in raw milk, and, thus, pose a different risk related to the consumption of raw milk (34). Raw milk offers ideal growth conditions for bacteria due to its rich nutrients, neutral pH, and high water activity but also exposes bacteria to environmental stresses: rich competing microbiota, bactericidal compounds, and cold storage (34, 35). Therefore, inability to proliferate under cold conditions and sensitivity to toxic derivatives formed by oxygen may hamper the survival of C. jejuni in raw milk (34).

Last, we investigated on-farm risk factors that could have affected milk contamination by STEC bacteria, as indicated by the presence of *stx*. Results suggest the desired effect of cleansing as a control measure. Effect of culling could be explained by variation in the fecal shedding of STEC between animals and over time, which has been studied extensively (reviewed in reference 24). Culling of a supershedder could reduce environmental contamination and, thereby, reduce the contamination pressure on milk (12). Pasturing could decrease shedding by shifting the diet from concentrated feed to fresh grass, as concentrated feed has been reported to increase STEC shedding (24). Alternatively, lower animal density on pastures could reduce udder contamination, thus reducing the contamination pressure on milk. Average outdoor temperature was observed to increase milk contamination, concordantly with the observations of higher STEC prevalence during warm months (24). With the limited data set used in our study, the results can be regarded as preliminary and should be verified with more data.

In conclusion, our study suggested persistence of STEC O157:H7 in the three Finnish herds for up to 12 months. Although the milk produced by the farms had good hygienic quality based on total bacterial counts, STEC contamination occurred occasionally during milking, especially at times when fecal shedding was detected. STEC was detected more frequently from milk filters than from five subsamples of milk, suggesting that sampling could be targeted to milk filters, reducing the number of required subsamples and, subsequently, analysis costs. Despite C. jejuni being prevalent in cattle, representing both persistent and sporadic strains, almost none could be detected from milk or milk filters. Lack of detection was probably due to poor survival of C. jejuni in these sample matrices. Therefore, analysis of milk or milk filters may poorly indicate the risk of campylobacteriosis related to the consumption of raw milk, especially if laboratory analyses are delayed after sampling. This should be considered in the planning of a sampling regime for monitoring. Furthermore, detection of various C. jejuni strains and highly pathogenic serogroups of STEC in the Finnish herds implies that categorization of farms as positive or negative may be arbitrary for targeted risk management. Instead, cost-effective on-farm hygienic practices should be promoted on all farms that produce raw drinking milk to reduce the contamination pressure on milk although the practices alone cannot prevent contamination. Because pathogen contamination occurred despite rigorous on-farm hygienic measures and because pathogen detection from milk and milk filters appeared challenging, the health risks of raw milk can effectively be avoided only by heat treatment of the milk before consumption.

## MATERIALS AND METHODS

### Farms.

Three Finnish dairy farms were recruited to the study on the basis that fecal samples of their cattle had previously tested positive for both STEC O157:H7 and C. jejuni. Before this study, STEC O157:H7 had been detected over 2 years previously on farm 1 (in October 2011 and January 2012), 4 months previously on farm 2 (in October 2013), and 2 months previously on farm 3 (in December 2013). Since the first detection of STEC O157:H7, the farms had followed national policies by implementing rigorous hygienic measures aimed at reducing on-farm transmission of this pathogen. These measures included continual disinfection of drinking and feeding troughs, along with enhanced hygiene during milking and handling of feed and manure. All farms were located in southern Finland and housed milking cows in warm free-stall barns. The cattle were pastured in summer (fenced off from natural waters) and reared indoors in winter. All new animal material was raised and silage was produced on the farms. Farm 1 housed 30 cows for pipeline milking in a parlor, whereas farms 2 and 3 both housed 60 cows in an automated milking system.

### Sampling, sample handling, and test portions.

Each farm was sampled for a year between January 2014 and June 2015: 52 to 53 times for milk and milk filters and 11 times for feces, drinking troughs, and drinking water. All of the samples were chilled immediately after collection and dispatched to the laboratory. Laboratory analyses were initiated as soon as possible, usually within 24 h of sampling. As an exception, milk filters were stored on farms after collection for up to 48 h, moistened with buffered peptone water, and dispatched to the laboratory with the milk samples. Sample containers were filled, or airtight sample bags were used to avoid drying and extra air space, detrimental for the survival of *Campylobacter*. All samples were examined in the national reference laboratory for STEC and thermophilic *Campylobacter* in food.

Milk samples (2 liters) were collected from the bulk tanks after 2 days of milk accumulation, before the tanks were voided. Milk was examined as five subsamples of 25 ml. All replaceable filters of the milking machines were collected during the same milk accumulation period, with 3 to 10 filters per sampling. Milk filters were longitudinally halved for STEC and *Campylobacter* analyses, and one to three halves were enriched as a sample in 225 or 450 ml of broth, depending on their size.

Freshly voided feces of 5 to 10 animals were pooled into one fecal sample according to the contemporary sampling regime of the Finnish monitoring program for STEC (36). Fecal samples were taken separately from milking cows and juvenile cattle in the barn and examined as 10-g test portions. Drinking troughs were sampled with duplicate sponge swabs (Polywipe; Medical Wire and Equipment, Corsham, Wiltshire, UK), and duplicates were divided between STEC and *Campylobacter* analyses. A single swab from the drinking troughs was enriched in 90 ml of broth. Samples from drinking water (8 liters), both in the barn and on the pastures, were taken if the water was sourced from a private supply, as was the case on farm 1. Drinking water was passed through 0.45-μm-pore-size membrane filters (GN-6 Metricel Membrane; Pall Corporation, Ann Arbor, MI, USA) as two subsamples of 4 liters, and the filters were halved for STEC and *Campylobacter* analyses. Filter halves of a 4-liter subsample were enriched in 100 ml or (for three or more halves) in 225 ml of broth.

### PCR screening and culture of STEC from milk and milk filters.

Milk and milk filters were analyzed for STEC according to International Organization for Standardization technical specification (ISO/TS) 13136:2012 (37). After enrichment in modified tryptone soya broth supplemented with 12 mg/liter acriflavine, milk and filter samples were simultaneously subjected to DNA extraction and immunomagnetic separation (IMS). DNA was extracted from 1 ml (MasterPure kit; Epicentre, Madison, WI, USA) or 100 μl (iQ-Check STEC kit; Bio-Rad, Marnes-la-Coquette, France) of enriched sample broth and screened for the virulence genes *stx* and *eae* using real-time PCR (TaqMan ISO assay [Life Technologies/Thermo Fisher Scientific, Carlsbad, CA, USA] or iQ-Check STEC kit). To avoid PCR signal interference, milk fat and acriflavine-containing broth were carefully removed before DNA extraction by pipetting and centrifugation. PCR inhibition was monitored with an internal amplification control. A minimum of one sample positive for *stx* and *eae* per sampling was further analyzed for serogroups O157:H7, O26, O103, O145, O111, O121, and O45 using real-time PCR (iQ-Check STEC kit).

IMS (Dynabeads anti-E. coli O157; Life Technologies/Thermo Fisher Scientific, Oslo, Norway) was followed by plating onto selective agars: cefixime-tellurite sorbitol MacConkey agar with 5-bromo-4-chloro-3-indoxyl-β-d-glucuronide (Harlequin; Lab M, Lancashire, UK) and CHROMagar STEC (CHROMagar, Paris, France). These plates were used for STEC detection throughout the study unless mentioned otherwise. The plates were screened for typical O157 colonies that were sorbitol and β-glucuronidase negative. To detect other STEC serogroups than the typical O157, 10 μl of enriched sample was plated without IMS, and the plates were screened for typical non-O157 colonies that were sorbitol positive. Suspected non-O157 colonies were streaked onto CHROMagar STEC and screened for the virulence genes using real-time PCR.

### STEC culture from cattle feces, drinking troughs, and drinking water.

Fecal samples were cultured for E. coli O157 according to the method of ISO 16654:2001, with the exceptions of enrichment for 6 h and use of selective agars (38). Swabs from drinking troughs and drinking water were examined for STEC O157 according to ISO/TS 13136:2012, with the enrichment in modified tryptone soya broth supplemented with 16 mg/liter novobiocin (37). Swabs were enriched for 6 h as an exception from the standard method. The enriched samples were subjected to IMS and cultured for the detection of typical STEC O157 without PCR screening. If STEC serogroups other than O157 were detected in the PCR screening of milk or milk filters, feces were additionally examined for that serogroup with IMS in one or two samplings.

### Confirmation and characterization of STEC isolates.

Suspected STEC isolates were biochemically confirmed as E. coli, tested for O-antigen agglutination (E. coli O157 latex test kit [Oxoid, Thermo Fisher Scientific, Basingstoke, UK] or E. coli rabbit antisera for O26, O111, O104, O103, or O145 [SSI Diagnostica, Hillerød, Denmark]), and examined for the presence of *stx*_1_, *stx*_2_, *eae*, and *hlyA* by conventional multiplex PCR (39). *stx*-positive isolates were subtyped using PFGE with XbaI digestion (40). Subtyping was performed for two isolates per positive milk, milk filter, or swab sample and one isolate per positive fecal sample. Gel images were analyzed using BioNumerics software (version 6.6; Applied Maths, Sint-Martens-Latem, Belgium). In PFGE fingerprints, a difference of one or more bands was designated a different pulsotype. Similarities in PFGE fingerprints were calculated using the Dice coefficient with 1.5% optimization and 1.0% tolerance.

### Analyses for C. jejuni.

Feces, milk, milk filters, and swabs from drinking troughs were cultured for thermophilic *Campylobacter* spp. by ISO 10272-1:2006 and drinking water by ISO 17995:2005, both methods with the exception of enrichment for 24 h (41, 42). Species of suspect *Campylobacter* isolates were identified biochemically or by matrix-assisted laser desorption ionization–time of flight mass spectrometry (MALDI Biotyper, reference library version 4.0.0.1, 5,627 main spectra libraries [Bruker Daltonik, Bremen, Germany]). C. jejuni isolates were subtyped using PFGE with SmaI digestion or, if nondigestible by SmaI, with KpnI (43). Isolates were selected for PFGE, and data were analyzed as described for STEC but with 0.5% optimization and 1% tolerance.

### Whole-genome sequencing, assembly, and *in silico* typing.

Based on pulsotypes, representative isolates were further selected for whole-genome sequencing. Altogether, sequences were obtained for 32 STEC O157, 5 STEC non-O157, and 36 C. jejuni isolates (see Table S1 and S2 in the supplemental material). After DNA extraction (PureLink genomic DNA minikit [Life Technologies/Thermo Fisher Scientific, Carlsbad, CA, USA] or DNeasy blood and tissue kit [Qiagen, Hilden, Germany]), genomic DNA libraries were prepared (Nextera XT or Nextera Flex kit; Illumina, San Diego, CA, USA) and subjected to paired-end sequencing (250-bp reads) on a HiSeq or MiSeq (Illumina) platform. Reads were further subjected to quality control, *de novo* assembly, and MLST by INNUca pipeline (version 3.1) (44). MLST types were derived from the PubMLST website (https://pubmlst.org/) (45–47). *In silico* serotypes, virulence genes for pathotyping E. coli, and *stx* subtypes were obtained for STEC isolates by reads mapping against the reference genes, respectively, using seq_typing (version 0.1), patho_typing (version 0.3), and ReMatCh (version 3.2) with the *stx* subtype reference sequences from the VirulenceFinder database (48–52). ReMatCh was run with the following initial parameters: minGeneCoverage, 100; minGeneIdentity, 100; minCovPresence, 1; minCovCall, 1. Manual curation of the positive hits to resolve presumably cross-reactant subtypes was then performed. Clade typing of STEC O157 isolates was performed as originally defined by Manning et al. (15), using eight definitive SNP positions according to Yokoyama et al. (53). Reads were first mapped against eight reference genes from the Sakai strain (GenBank accession no. NC_002695.1) (54, 55) by ReMatCh with default parameters, and definitive SNPs were then determined from the resulting alignments.

### Whole-genome multilocus sequence typing.

To assess genomic variation of the data sets in a wider or global context, draft genome assemblies were subjected to wgMLST using chewBBACA software (version 2.0.8) with the chewBBACA schemas for E. coli and C. jejuni (16, 17, 56, 57). Resulting allelic profiles were concatenated with the profiles representing the same MLST ST from the INNUENDO database (16, 17). After the core loci were extracted, PWDs were calculated and used to infer a minimum spanning tree in PHYLOViZ Online (https://online.phyloviz.net/) that uses the goeBURST algorithm (58).

Analyses were performed for four data sets, as follows: (i) allelic profiles of 32 STEC O157:H7 isolates originating from all three farms were compared with 482 ST-11 profiles from the INNUENDO database (Data Set S1); (ii) allelic profiles of six C. jejuni isolates from the three farms were compared with 436 ST-45 profiles from the INNUENDO database (Data Set S2); (iii) allelic profiles of three C. jejuni isolates from farm 1 were compared with four profiles from the database, all representing ST-1080 (Data Set S3); (iv) allelic profiles of five C. jejuni isolates from farm 3 were compared with 66 ST-883 profiles from the database (Data Set S4).

### Phylogenomics of STEC O157:H7 isolates.

Based on PWDs in wgMLST, the closest foreign isolate to STEC O157:H7 farm isolates was selected as an outgroup for further analyses. Its assembled genome sequence was obtained from the EnteroBase database (accession number ESC_FA0769AA) and reads from the European Nucleotide Archive (accession no. SRR4787064) (57, 59). The assembled genome sequences of 32 STEC O157:H7 farm isolates and the outgroup were annotated with Prokka (version 1.12), followed by pangenome analysis with Roary (version 3.8.0) to select an in-group reference genome with the highest number of coding sequences (60, 61).

SNPs were then called from the sequencing reads of 31 genomes against the assembled reference genome (Ec_Farm2_2014-03_C1), and core SNPs were extracted using Snippy (version 4.0-dev) (62). The analysis was performed both with and without the outgroup (ESC_FA0769AA). The full sequence alignment of core SNPs and invariant sites was then analyzed for recombination using Gubbins (version 2.3.1), and recombinant regions were masked from the alignment by maskrc-svg (version 0.4) (63, 64). From the recombination-free alignment, a maximum likelihood tree was constructed using IQ-TREE (version 1.5.5) with automatic model selection and both the SH-aLRT test and UFBoot bootstrapping with 1,000 replicates (65–67). An automatically selected substitution model, K3Pu+R7, assumed three substitution types, unequal base frequencies, and a free rate of heterogeneity across sites. To test the validity of the molecular clock assumption, a temporal signal was investigated from the resulting phylogeny by root-to-tip analysis in TempEst (version 1.5.1) with the best-fit root option (68).

### Analysis of risk factors for milk contamination by *stx*.

Simultaneously with each milk sampling, the farmers filled out a questionnaire on deviations from normal farm practices during the preceding week. The questionnaire data consisted of 26 questions (i.e., variables), each with 157 binary answers (i.e., observations for a single variable; presence/absence data, three farms for 52 to 53 weeks). Excluding missing data, each question contained 152 binary answers. Questions were excluded from the analysis based on: (i) zero inflation with less than 5% of presence data (16 questions excluded), (ii) biological irrelevance to milk contamination (3 questions excluded: data on juvenile cattle and on unspecified farm visitors), (iii) heterogeneous reporting practices between the farms (1 question excluded: changes in staff), or (iv) notable collinearity with another variable (1 question excluded: questionnaire data on udder health).

In addition to the questionnaire, total bacterial counts (variable Bact) and somatic cell counts (variable Cell) were included in the analysis as indicators for milk hygiene and udder health, respectively. These results had been obtained by dairy laboratories in separate samplings from our study, respectively, using flow cytometry (BactoScan FC; Foss, Hillerød, Denmark) and fluoro-opto-electronic methods (69). Therefore, respectively, 89 (57%) of Bact and 93 (59%) of Cell results were analyzed from the same bulk tank content by the dairy laboratories and this study. The missing values were either (i) replaced with plausible values from temporally close results (49 Bact and 57 Cell values) or, where these were unavailable, (ii) multiply imputed within the model from a distribution exploiting the observed values. One large value was observed for both variables Bact and Cell, but their removal as outliers could not be justified biologically.

To include meteorological variables, weather observations were retrieved from the nearest weather station of each farm, considering distance to the coast (70). Temperature and humidity were hypothesized to mediate milk contamination by affecting conditions for bacterial survival and growth in the barn and on pastures. Number of rainy days was used as an indicator for humidity because of its robustness to seasonal effect.

Altogether, nine explanatory variables (Table 5) were included in the model after data exploration (71). Continuous variables (Bact, Cell, and Temp) were standardized ([*x_i_* – μ]/σ) before the analysis (model M1):

$$\text{logit}\left(\theta_{ij}\right)\;=\;\beta_{0j}\;+\;\beta_{1}{\text{Sale}}_{i}\;+\;\beta_{2}{\text{Cleanse}}_{i}\;+\;\beta_{3}{\text{Feed}}_{i}\;+\;\beta_{4}{\text{Pastured}}_{i}\;+\;\beta_{5}{\text{Maint}}_{i}\;+\;\beta_{6}{\text{Rain}}_{i}\;+\;\beta_{7}{\text{Temp}}_{i}\;+\;\beta_{8}{\text{Bact}}_{i}\;+\;\beta_{9}{\text{Cell}}_{i}$$

$$y_{ij}^{}|\theta_{ij}∼\text{Binomial}\left(n_{ij},\theta_{ij}\right)$$

where *y_ij_* is the number of *stx*-positive milk subsamples in sampling *i* of farm *j*, *n_ij_* is the number of milk subsamples in sampling *i* of farm *j*, and θ*_ij_* is the proportion of *stx*-positive milk subsamples in sampling *i* of farm *j*, with the following uninformative priors:

$$p\left(\beta_{0j}\right)=N\left(0,10^{3}\right),j=1,2,3$$

$$p\left(\beta_{k}\right)=N\left(0,10^{3}\right),k=1,\ldots ,9$$

We determined for missing data imputation Sale*_ij_* ~ Bernoulli(*p*_1_), Cleanse*_ij_* ~ Bernoulli(*p*_2_), Feed*_ij_* ~ Bernoulli(*p*_3_), Pastured*_ij_* ~ Bernoulli(*p*_4_), Maint*_ij_* ~ Bernoulli(*p*_5_), Bact*_ij_* ~ *N*(μ_1_,τ_1_), and Cell*_ij_* ~ *N*(μ_2_,τ_2_), with the following uninformative priors:

$$p\left(p_{l}\right)=Beta\left(1,\;1\right),l=1,\ldots ,5$$

$$p\left(\mu_{m}\right)=N\left(0,10^{3}\right),m=1,2$$

$$p\left(\tau_{m}\right)=Gamma\left(10^{-2},10^{-2}\right),m=1,2$$

Data were analyzed using R software (version 3.4.4) and JAGS software (version 4.3.0) via the rjags package (version 4–6) (72–74). Markov chain Monte Carlo simulations were run using 10,000 iterations in two chains with thinning of 2 and adaptation of 200 iterations. Convergence and auto-correlation were checked from the resulting chains. Posterior probabilities, *P*(β*_k_* > 0), and 95% credibility intervals were calculated for β*_k_*. Explanatory variables were interpreted to increase milk contamination at a *P*(β*_k_* > 0) of >0.95, to decrease milk contamination at a *P*(β*_k_* > 0) of <0.05, and to have no effect on milk contamination at a *P*(β*_k_* > 0) of ≥0.05 and ≤0.95.

### Data availability.

*De novo* genome assemblies (Data Set S5) and data and code for R are available from the Zenodo repository (https://doi.org/10.5281/zenodo.1467141). Whole-genome sequencing reads were submitted to the European Nucleotide Archive under project accession numbers PRJEB28441 and PRJEB27020 (Tables S1 and S2).

## Supplementary Material

Supplemental file 1

Supplemental file 2

## References

[1] European Food Safety Authority. 2015 Scientific opinion on the public health risks related to the consumption of raw drinking milk. EFSA J 13:3940. doi:10.2903/j.efsa.2015.3940. PMC13189509 42169838

[2] Mungai EA, Behravesh CB, Gould LH 2015 Increased outbreaks associated with nonpasteurized milk, United States, 2007–2012. Emerg Infect Dis 21:119–122. doi:10.3201/eid2101.140447. 25531403 PMC4285278

[3] Oliver SP, Boor KJ, Murphy SC, Murinda SE 2009 Food safety hazards associated with consumption of raw milk. Foodborne Pathog Dis 6:793–806. doi:10.1089/fpd.2009.0302. 19737059

[4] Farrokh C, Jordan K, Auvray F, Glass K, Oppegaard H, Raynaud S, Thevenot D, Condron R, De Reu K, Govaris A, Heggum K, Heyndrickx M, Hummerjohann J, Lindsay D, Miszczycha S, Moussiegt S, Verstraete K, Cerf O 2013 Review of Shiga-toxin-producing *Escherichia coli* (STEC) and their significance in dairy production. Int J Food Microbiol 162:190–212. doi:10.1016/j.ijfoodmicro.2012.08.008. 22939912

[5] Hakkinen M, Hänninen ML 2009 Shedding of *Campylobacter* spp. in Finnish cattle on dairy farms. J Appl Microbiol 107:898–905. doi:10.1111/j.1365-2672.2009.04269.x. 19486409

[6] Ruusunen M, Salonen M, Pulkkinen H, Huuskonen M, Hellström S, Revez J, Hänninen ML, Fredriksson-Ahomaa M, Lindström M 2013 Pathogenic bacteria in Finnish bulk tank milk. Foodborne Pathog Dis 10:99–106. doi:10.1089/fpd.2012.1284. 23373473

[7] Christidis T, Pintar KDM, Butler AJ, Nesbitt A, Thomas MK, Marshall B, Pollari F 2016 *Campylobacter* spp. prevalence and levels in raw milk: a systematic review and meta-analysis. J Food Prot 79:1775–1783. doi:10.4315/0362-028X.JFP-15-480. 28221843

[8] Van Kessel JA, Karns JS, Lombard JE, Kopral CA 2011 Prevalence of *Salmonella enterica*, *Listeria monocytogenes*, and *Escherichia coli* virulence factors in bulk tank milk and in-line filters from U.S. dairies. J Food Prot 74:759–768. doi:10.4315/0362-028X.JFP-10-423. 21549046

[9] Bianchini V, Borella L, Benedetti V, Parisi A, Miccolupo A, Santoro E, Recordati C, Luini M 2014 Prevalence in bulk tank milk and epidemiology of *Campylobacter jejuni* in dairy herds in Northern Italy. Appl Environ Microbiol 80:1832–1837. doi:10.1128/AEM.03784-13. 24413598 PMC3957646

[10] Murinda SE, Nguyen LT, Ivey SJ, Gillespie BE, Almeida RA, Draughon FA, Oliver SP 2002 Prevalence and molecular characterization of *Escherichia coli* O157:H7 in bulk tank milk and fecal samples from cull cows: a 12-month survey of dairy farms in East Tennessee. J Food Prot 65:752–759. doi:10.4315/0362-028X-65.5.752. 12030284

[11] McAuley CM, McMillan K, Moore SC, Fegan N, Fox EM 2014 Prevalence and characterization of foodborne pathogens from Australian dairy farm environments. J Dairy Sci 97:7402–7412. doi:10.3168/jds.2014-8735. 25282417

[12] Murphy BP, McCabe E, Murphy M, Buckley JF, Crowley D, Fanning S, Duffy G 2016 Longitudinal study of two Irish dairy herds: low numbers of Shiga toxin-producing *Escherichia coli* O157 and O26 super-shedders identified. Front Microbiol 7:1850. doi:10.3389/fmicb.2016.01850. 27917164 PMC5114295

[13] Artursson K, Schelin J, Lambertz ST, Hansson I, Engvall EO 2018 Foodborne pathogens in unpasteurized milk in Sweden. Int J Food Microbiol 284:120–127. doi:10.1016/j.ijfoodmicro.2018.05.015. 29887505

[14] Croxen MA, Law RJ, Scholz R, Keeney KM, Wlodarska M, Finlay BB 2013 Recent advances in understanding enteric pathogenic *Escherichia coli*. Clin Microbiol Rev 26:822–880. doi:10.1128/CMR.00022-13. 24092857 PMC3811233

[15] Manning SD, Motiwala AS, Springman AC, Qi W, Lacher DW, Ouellette LM, Mladonicky JM, Somsel P, Rudrik JT, Dietrich SE, Zhang W, Swaminathan B, Alland D, Whittam TS 2008 Variation in virulence among clades of *Escherichia coli* O157:H7 associated with disease outbreaks. Proc Natl Acad Sci U S A 105:4868. doi:10.1073/pnas.0710834105. 18332430 PMC2290780

[16] Rossi M, Silva MS, Ribeiro-Gonçalves BF, Silva DN, Machado MP, Oleastro M, Borges V, Isidro J, Viera L, Halkilahti J, Jaakkonen A, Palma F, Salmenlinna S, Hakkinen M, Garaizar J, Bikandi J, Hilbert F, Carriço JA 2018 INNUENDO whole genome and core genome MLST schemas and datasets for *Escherichia coli*. Zenodo https://zenodo.org/record/1323690.

[17] Rossi M, Silva MS, Ribeiro-Gonçalves BF, Silva DN, Machado MP, Oleastro M, Borges V, Isidro J, Viera L, Barker Dor Llarena AK, Halkilahti J, Jaakkonen A, Kivistö R, Kovanen S, Nieminen T, Hänninen ML, Salmenlinna S, Hakkinen M, Garaizar J, Bikandi J, Hilbert F, Taboada EN, Carriço JA 2018 INNUENDO whole genome and core genome MLST schemas and datasets for *Campylobacter jejuni*. Zenodo https://zenodo.org/record/1322564.

[18] Adam K, Brülisauer F 2010 The application of food safety interventions in primary production of beef and lamb: a review. Int J Food Microbiol 141:S43–S52. doi:10.1016/j.ijfoodmicro.2009.12.020. 20097438

[19] Jaakkonen A, Salmenlinna S, Rimhanen-Finne R, Lundström H, Heinikainen S, Hakkinen M, Hallanvuo S 2017 Severe outbreak of sorbitol-fermenting *Escherichia coli* O157 via unpasteurized milk and farm visits, Finland 2012. Zoonoses Public Health 64:468–475. doi:10.1111/zph.12327. 28045227

[20] Martínez-Castillo A, Muniesa M 2014 Implications of free Shiga toxin-converting bacteriophages occurring outside bacteria for the evolution and the detection of Shiga toxin-producing *Escherichia coli*. Front Cell Infect Microbiol 4:46. doi:10.3389/fcimb.2014.00046. 24795866 PMC3997033

[21] Food and Agriculture Organization, World Health Organization. 2018 Shiga toxin-producing Escherichia coli (STEC) and food: attribution, characterization, and monitoring. Microbiological Risk Assessment Series 31 World Health Organization, Geneva, Switzerland.

[22] Vanaja SK, Springman AC, Besser TE, Whittam TS, Manning SD 2010 Differential expression of virulence and stress fitness genes between *Escherichia coli* O157:H7 strains with clinical or bovine-biased genotypes. Appl Environ Microbiol 76:60–68. doi:10.1128/AEM.01666-09. 19880650 PMC2798638

[23] Worley JN, Flores KA, Yang X, Chase JA, Cao G, Tang S, Meng J, Atwill ER 2017 Prevalence and genomic characterization of *Escherichia coli* O157:H7 in cow-calf herds throughout California. Appl Environ Microbiol 83:e00734-17. doi:10.1128/AEM.00734-17. 28550057 PMC5541215

[24] Berry ED, Wells JE 2010 *Escherichia coli* O157:H7: recent advances in research on occurrence, transmission, and control in cattle and the production environment. Adv Food Nutr Res 60:67–117. doi:10.1016/S1043-4526(10)60004-6. 20691954

[25] Sheppard SK, Maiden MCJ 2015 The evolution of *Campylobacter jejuni* and *Campylobacter coli*. Cold Spring Harb Perspect Biol 7:a018119. doi:10.1101/cshperspect.a018119. 26101080 PMC4526750

[26] Weis AM, Storey DB, Taff CC, Townsend AK, Huang BC, Kong NT, Clothier KA, Spinner A, Byrne BA, Weimer BC 2016 Genomic comparison of *Campylobacter* spp. and their potential for zoonotic transmission between birds, primates, and livestock. Appl Environ Microbiol 82:7165–7175. doi:10.1128/AEM.01746-16. 27736787 PMC5118927

[27] Llarena AK, Skarp-de Haan CPA, Rossi M, Hänninen ML 2015 Characterization of the *Campylobacter jejuni* population in the barnacle geese reservoir. Zoonoses Public Health 62:209–221. doi:10.1111/zph.12141. 24948379

[28] Dearlove BL, Cody AJ, Pascoe B, Méric G, Wilson DJ, Sheppard SK 2016 Rapid host switching in generalist *Campylobacter* strains erodes the signal for tracing human infections. ISME J 10:721–729. doi:10.1038/ismej.2015.149. 26305157 PMC4677457

[29] Bronowski C, James CE, Winstanley C 2014 Role of environmental survival in transmission of *Campylobacter jejuni*. FEMS Microbiol Lett 356:8–19. doi:10.1111/1574-6968.12488. 24888326

[30] Yahara K, Méric G, Taylor AJ, de Vries SPW, Murray S, Pascoe B, Mageiros L, Torralbo A, Vidal A, Ridley A, Komukai S, Wimalarathna H, Cody AJ, Colles FM, McCarthy N, Harris D, Bray JE, Jolley KA, Maiden MCJ, Bentley SD, Parkhill J, Bayliss CD, Grant A, Maskell D, Didelot X, Kelly DJ, Sheppard SK 2017 Genome-wide association of functional traits linked with *Campylobacter jejuni* survival from farm to fork. Environ Microbiol 19:361–380. doi:10.1111/1462-2920.13628. 27883255

[31] Giacometti F, Serraino A, Finazzi G, Daminelli P, Losio MN, Bonilauri P, Arrigoni N, Garigliani A, Mattioli R, Alonso S, Piva S, Florio D, Riu R, Zanoni RG 2012 Foodborne pathogens in in-line milk filters and associated on-farm risk factors in dairy farms authorized to produce and sell raw milk in Northern Italy. J Food Prot 75:1263–1269. doi:10.4315/0362-028X.JFP-12-028. 22980010

[32] Teunis P, Van den Brandhof W, Nauta M, Wagenaar J, Van den Kerkhof H, Van Pelt W 2005 A reconsideration of the *Campylobacter* dose–response relation. Epidemiol Infect 133:583–592. doi:10.1017/S0950268805003912. 16050502 PMC2870284

[33] Jarvis B, Wilrich C, Wilrich PT 2018 Reconsideration of the derivation of most probable numbers, their standard deviations, confidence bounds and rarity values. J Appl Microbiol 119:905–905. doi:10.1111/jam.12901. 26297181

[34] Doyle MP, Roman DJ 1982 Prevalence and survival of *Campylobacter jejuni* in unpasteurized milk. Appl Environ Microbiol 44:1154–1158. 6897503 10.1128/aem.44.5.1154-1158.1982 PMC242162

[35] Quigley L, O'Sullivan O, Stanton C, Beresford TP, Ross RP, Fitzgerald GF, Cotter PD 2013 The complex microbiota of raw milk. FEMS Microbiol Rev 37:664–698. doi:10.1111/1574-6976.12030. 23808865

[36] Ministry of Agriculture and Forestry of Finland. 2006 Regulation no. 24/EEO/2006. Maa- ja metsätalousministeriön asetus nautojen EHEC-tutkimuksista teurastamossa ja pitopaikassa. (In Finnish.) https://mmm.fi/documents/1410837/1818689/24EEO2006.pdf.

[37] International Organization for Standardization. 2012 ISO/TS 13136:2012. Microbiology of food and animal feed—real-time polymerase chain reaction (PCR)-based method for the detection of food-borne pathogens – Horizontal method for the detection of Shiga toxin-producing *Escherichia coli* (STEC) and the determination of O157, O111, O26, O103 and O145 serogroups. International Organization for Standardization, Geneva, Switzerland.

[38] International Organization for Standardization. 2001 ISO 16654:2001. Microbiology of food and animal feeding stuffs—horizontal method for the detection of *Escherichia coli* O157. International Organization for Standardization, Geneva, Switzerland. 10.1016/j.ijfoodmicro.2018.05.005 29778498

[39] Paton AW, Paton JC 1998 Detection and characterization of Shiga toxigenic *Escherichia coli* by using multiplex PCR assays for *stx*_1_, *stx*_2_, *eaeA*, enterohemorrhagic *E. coli hlyA, rfb*_O111_, and *rfb*_O157_. J Clin Microbiol 36:598–602. 9466788 10.1128/jcm.36.2.598-602.1998 PMC104589

[40] Centers for Disease Control and Prevention. 2013 Standard operating procedure for PulseNet PFGE of *Escherichia coli* O157:H7, *Escherichia coli* non-O157 (STEC), *Salmonella* serotypes, *Shigella sonnei* and *Shigella flexneri*. Centers for Disease Control and Prevention, Atlanta, GA.

[41] International Organization for Standardization. 2006 ISO 10272-1:2006. Microbiology of food and animal feeding stuffs—horizontal method for detection and enumeration of *Campylobacter* spp. Part 1: detection method. International Organization for Standardization, Geneva, Switzerland.

[42] International Organization for Standardization. 2005 ISO 17995:2005. Water quality—detection and enumeration of thermotolerant *Campylobacter* species. International Organization for Standardization, Geneva, Switzerland.

[43] Centers for Disease Control and Prevention 2013 Standard operating procedure for PulseNet PFGE of *Campylobacter jejuni*. Centers for Disease Control and Prevention, Atlanta, GA.

[44] Machado MP, Halkilahti J, Jaakkonen A, Silva DN, Mendes I, Nalbantoglu Y, Borges V, Ramirez M, Rossi M, Carriço JA 2017 INNUca, version 3.1. https://github.com/B-UMMI/INNUca.

[45] Jolley KA, Bray JE, Maiden MCJ 2018 Open-access bacterial population genomics: BIGSdb software, the PubMLST.org website and their applications. Wellcome Open Res 3:124. doi:10.12688/wellcomeopenres.14826.1. 30345391 PMC6192448

[46] Wirth T, Falush D, Lan R, Colles F, Mensa P, Wieler LH, Karch H, Reeves PR, Maiden MCJ, Ochman H, Achtman M 2006 Sex and virulence in *Escherichia coli*: an evolutionary perspective. Mol Microbiol 60:1136–1151. doi:10.1111/j.1365-2958.2006.05172.x. 16689791 PMC1557465

[47] Dingle KE, Colles FM, Wareing DRA, Ure R, Fox AJ, Bolton FE, Bootsma HJ, Willems RJL, Urwin R, Maiden MCJ 2001 Multilocus sequence typing system for *Campylobacter jejuni*. J Clin Microbiol 39:14–23. doi:10.1128/JCM.39.1.14-23.2001. 11136741 PMC87672

[48] Machado MP 2018 seq_typing, version 0.1. https://github.com/B-UMMI/seq_typing.

[49] Machado MP 2017 patho_typing, version 0.3. https://github.com/B-UMMI/patho_typing.

[50] Machado MP 2017 ReMatCh, version 3.2. https://github.com/B-UMMI/ReMatCh.

[51] Center for Genomic Epidemiology. 2014 VirulenceFinder database for *Escherichia coli*. https://bitbucket.org/genomicepidemiology/virulencefinder_db. Accessed 26 June 2018.

[52] Joensen KG, Scheutz F, Lund O, Hasman H, Kaas RS, Nielsen EM, Aarestrup FM 2014 Real-time whole-genome sequencing for routine typing, surveillance, and outbreak detection of verotoxigenic *Escherichia coli*. J Clin Microbiol 52:1501–1510. doi:10.1128/JCM.03617-13. 24574290 PMC3993690

[53] Yokoyama E, Hirai S, Hashimoto R, Uchimura M 2012 Clade analysis of enterohemorrhagic *Escherichia coli* serotype O157:H7/H- strains and hierarchy of their phylogenetic relationships. Infect Genet Evol 12:1724–1728. doi:10.1016/j.meegid.2012.07.003. 22846398

[54] Hayashi T, Makino K, Ohnishi M, Kurokawa K, Ishii K, Yokoyama K, Han CG, Ohtsubo E, Nakayama K, Murata T, Tanaka M, Tobe T, Iida T, Takami H, Honda T, Sasakawa C, Ogasawara N, Yasunaga T, Kuhara S, Shiba T, Hattori M, Shinagawa H 2001 Complete genome sequence of enterohemorrhagic *Escherichia coli* O157:H7 and genomic comparison with a laboratory strain K-12. DNA Res 8:11–22. doi:10.1093/dnares/8.1.11. 11258796

[55] Hayashi T, Makino K, Ohnishi M, Kurokawa K, Ishii K, Yokoyama K, Han CG, Ohtsubo E, Nakayama K, Murata T, Tanaka M, Tobe T, Iida T, Takami H, Honda T, Sasakawa C, Ogasawara N, Yasunaga T, Kuhara S, Shiba T, Hattori M, Shinagawa H 2017 Data from “Complete genome sequence of enterohemorrhagic *Escherichia coli* O157:H7 and genomic comparison with a laboratory strain K-12.” GenBank database https://www.ncbi.nlm.nih.gov/nuccore/NC_002695.1 (accession no. NC_002695.1). 10.1093/dnares/8.1.11 11258796

[56] Silva M, Machado MP, Silva DN, Rossi M, Moran-Gilad J, Santos S, Ramirez M, Carriço JA 2018 chewBBACA: a complete suite for gene-by-gene schema creation and strain identification. Microb Genom 4:e000166. doi:10.1099/mgen.0.000166. PMC5885018 29543149

[57] Alikhan N, Zhou Z, Sergeant MJ, Achtman M 2018 A genomic overview of the population structure of *Salmonella*. PLoS Genet 14:e1007261. doi:10.1371/journal.pgen.1007261. 29621240 PMC5886390

[58] Francisco AP, Bugalho M, Ramirez M, Carriço JA 2009 Global optimal eBURST analysis of multilocus typing data using a graphic matroid approach. BMC Bioinformatics 10:152. doi:10.1186/1471-2105-10-152. 19450271 PMC2705362

[59] Health Protection Agency. 2016 Data from “Routine surveillance of *E. coli* and *Shigella* by Public Health England”. European Nucleotide Archive https://www.ebi.ac.uk/ena/data/view/SRR4787064 (accession no. SRR4787064).

[60] Seemann T 2014 Prokka: rapid prokaryotic genome annotation. Bioinformatics 30:2068–2069. doi:10.1093/bioinformatics/btu153. 24642063

[61] Page AJ, Cummins CA, Hunt M, Wong VK, Reuter S, Holden MTG, Fookes M, Falush D, Keane JA, Parkhill J 2015 Roary: rapid large-scale prokaryote pan genome analysis. Bioinformatics 31:3691–3693. doi:10.1093/bioinformatics/btv421. 26198102 PMC4817141

[62] Seemann T 2018 Snippy, version 4.0-dev. https://github.com/tseemann/snippy.

[63] Croucher NJ, Page AJ, Connor TR, Delaney AJ, Keane JA, Bentley SD, Parkhill J, Harris SR 2015 Rapid phylogenetic analysis of large samples of recombinant bacterial whole genome sequences using Gubbins. Nucleic Acids Res 43:e15. doi:10.1093/nar/gku1196. 25414349 PMC4330336

[64] Kwong J, Seemann T 2018 maskrc-svg, version 0.4. https://github.com/kwongj/maskrc-svg.

[65] Nguyen L, Schmidt HA, von Haeseler A, Minh BQ 2015 IQ-TREE: a fast and effective stochastic algorithm for estimating maximum-likelihood phylogenies. Mol Biol Evol 32:268–274. doi:10.1093/molbev/msu300. 25371430 PMC4271533

[66] Kalyaanamoorthy S, Minh BQ, Wong TKF, von Haeseler A, Jermiin LS 2017 ModelFinder: fast model selection for accurate phylogenetic estimates. Nat Methods 14:587–589. doi:10.1038/nmeth.4285. 28481363 PMC5453245

[67] Hoang DT, Chernomor O, von Haeseler A, Minh BQ, Vinh LS 2018 UFBoot2: improving the ultrafast bootstrap approximation. Mol Biol Evol 35:518–522. doi:10.1093/molbev/msx281. 29077904 PMC5850222

[68] Rambaut A, Lam TT, Max Carvalho L, Pybus OG 2016 Exploring the temporal structure of heterochronous sequences using TempEst (formerly Path-O-Gen). Virus Evol 2:vew007. doi:10.1093/ve/vew007. 27774300 PMC4989882

[69] International Organization for Standardization. 2006 ISO 13366-2:2006 (IDF 148-2:2006). Milk—enumeration of somatic cells. Part 2: guidance on the operation of fluoro-opto-electronic counters. International Organization for Standardization, Geneva, Switzerland.

[70] Finnish Meteorological Institute. 2015 Daily weather observations. https://en.ilmatieteenlaitos.fi/. Accessed 13 August 2015.

[71] Zuur AF, Ieno EN, Elphick CS 2010 A protocol for data exploration to avoid common statistical problems. Methods Ecol Evol 1:3–14. doi:10.1111/j.2041-210X.2009.00001.x.

[72] R Core Team. 2018 R: a language and environment for statistical computing. R Foundation for Statistical Computing, Vienna, Austria.

[73] Plummer M 2003 JAGS: a program for analysis of Bayesian graphical models using Gibbs sampling. *In* Hornik K, Leisch F, Zeileis A (ed), Proceedings of the 3rd International Workshop on Distributed Statistical Computing (DSC 2003). R Foundation for Statistical Computing, Vienna, Austria.

[74] Plummer M 2016 rjags: Bayesian graphical models using MCMC, version 4–6. R Foundation for Statistical Computing, Vienna, Austria. https://CRAN.R-project.org/package=rjags.

[75] Letunic I, Bork P 2016 Interactive tree of life (iTOL) v3: an online tool for the display and annotation of phylogenetic and other trees. Nucleic Acids Res 44:W242–W245. doi:10.1093/nar/gkw290. 27095192 PMC4987883

