# Supplementary material for: Longitudinal Study of Shiga Toxin-Producing Escherichia coli and Campylobacter jejuni on Finnish Dairy Farms and in Raw Milk
Source: Appl Environ Microbiol. 2019 Mar 22;85(7):e02910-18. doi: 10.1128/AEM.02910-18 (PMC6585499; doi:10.1128/AEM.02910-18)
Supplement: Supplemental file 1 [file AEM.02910-18-s0001.pdf]

TABLE S1 Whole-genome sequencing (WGS), serotyping, pathotyping, and *stx* subtyping of STEC isolates from three dairy farms, collected from October 2011 through May 2015. Data were submitted to the European Nucleotide Archive under the project accession no. PRJEB27020 ([a]) or PRJEB28441.

Run accession no.	Alias[b]	Serotype	Virulence[c]
ERR2834499	Ec_Farm1_2011-10_C1	O157:H7	*stx1a*, *stx2c*, *eae*, *hlyA*
ERR2834500	Ec_Farm1_2011-10_E1	O157:H7	*stx1a*, *stx2c*, *eae*, *hlyA*
ERR2834501	Ec_Farm1_2011-10_E2	O157:H7	*stx1a*, *stx2c*, *eae*, *hlyA*
ERR2834502	Ec_Farm1_2012-01_E1	O157:H7	*stx1a*, *stx2c*, *eae*, *hlyA*
ERR2834503	Ec_Farm1_2012-01_E2	O157:H7	*stx1a*, *stx2c*, *eae*, *hlyA*
ERR2834504	Ec_Farm1_2014-09_C1	O157:H7	*stx1a*, *stx2c*, *eae*, *hlyA*
ERR2834505	Ec_Farm1_2014-09_C2	O157:H7	*stx1a*, *stx2c*, *eae*, *hlyA*
ERR2602174[a]	Ec_Farm2_2013-10_C1	O157:H7	*stx1a*, *stx2c*, *eae*, *hlyA*
ERR2602173[a]	Ec_Farm2_2013-10_E1	O157:H7	*stx1a*, *stx2c*, *eae*, *hlyA*
ERR2834506	Ec_Farm2_2014-03_C1	O157:H7	*stx1a*, *stx2c*, *eae*, *hlyA*
ERR2834507	Ec_Farm2_2014-03_C2	O157:H7	*stx1a*, *stx2c*, *eae*, *hlyA*
ERR2602181[a]	Ec_Farm2_2014-03_C3	O157:H7	*stx1a*, *stx2c*, *eae*, *hlyA*
ERR2834508	Ec_Farm2_2014-03_E1	O157:H7	*stx1a*, *stx2c*, *eae*, *hlyA*
ERR2602180[a]	Ec_Farm2_2014-03_F1	O157:H7	*stx1a*, *stx2c*, *eae*, *hlyA*
ERR2602182[a]	Ec_Farm2_2014-04_C1	O157:H7	*stx1a*, *stx2c*, *eae*, *hlyA*
ERR2834509	Ec_Farm2_2014-04_C2	O157:H7	*stx1a*, *stx2c*, *eae*, *hlyA*
ERR2834510	Ec_Farm2_2014-05_C1	O157:H7	*stx1a*, *stx2c*, *eae*, *hlyA*
ERR2602184[a]	Ec_Farm2_2014-05_F1	O157:H7	*stx1a*, *stx2c*, *eae*, *hlyA*
ERR2834511	Ec_Farm2_2014-06_C1	O157:H7	*stx1a*, *stx2c*, *eae*, *hlyA*
ERR2602186[a]	Ec_Farm2_2014-06_C2	O157:H7	*stx1a*, *stx2c*, *eae*, *hlyA*
ERR2834512	Ec_Farm2_2014-06_F1	O157:H7	*stx1a*, *stx2c*, *eae*, *hlyA*
ERR2834513	Ec_Farm2_2014-06_F2	O157:H7	*stx1a*, *stx2c*, *eae*, *hlyA*
ERR2602187[a]	Ec_Farm2_2014-08_C1	O157:H7	*stx1a*, *stx2c*, *eae*, *hlyA*
ERR2834514	Ec_Farm2_2014-09_C1	O157:H7	*stx1a*, *stx2c*, *eae*, *hlyA*
ERR2602189[a]	Ec_Farm2_2014-09_F1	O157:H7	*stx1a*, *stx2c*, *eae*, *hlyA*
ERR2602175[a]	Ec_Farm3_2014-01_C1	O157:H7	*stx1a*, *stx2c*, *eae*, *hlyA*
ERR2602179[a]	Ec_Farm3_2014-02_C1	O157:H7	*stx1a*, *stx2c*, *eae*, *hlyA*
ERR2602178[a]	Ec_Farm3_2014-02_E1	O157:H7	*stx1a*, *stx2c*, *eae*, *hlyA*
ERR2602183[a]	Ec_Farm3_2014-05_C1	O157:H7	*stx1a*, *stx2c*, *eae*, *hlyA*
ERR2602185[a]	Ec_Farm3_2014-06_F1	O157:H7	*stx1a*, *stx2c*, *eae*, *hlyA*
ERR2602190[a]	Ec_Farm3_2014-07_C1	O157:H7	*stx1a*, *stx2c*, *eae*, *hlyA*
ERR2602191[a]	Ec_Farm3_2014-11_E1	O157:H7	*stx1a*, *stx2c*, *eae*, *hlyA*
ERR2834515	Ec_Farm2_2014-10_F1	O182:H25	*stx1a*, *eae*, *hlyA*
ERR2834516	Ec_Farm2_2015-05_C1	O15:H16	*stx2g*, *estIa*
ERR2834517	Ec_Farm3_2014-07_M1	O121:H19	*stx2a*, *eae*, *hlyA*
ERR2834518	Ec_Farm3_2014-10_C1	O84:H2	*stx2c*, *eae*, *hlyA*
ERR2834519	Ec_Farm3_2015-05_C1	O26:H11	*stx1a*, *eae*, *hlyA*

[b]Aliases indicate the following metadata for the isolates: *E. coli*, farm no., isolate collection time (yyyy-mm), source (C=cattle feces, E=farm environment, F=milk filter, M=bulk tank milk), and colony no.

[c]In addition to Shiga Toxin genes (*stx*), the isolates (except the O15:H16 isolate) harbored *eae* and *hlyA*, encoding for intimin and enterohemolysin, respectively. The O15:H16 isolate harbored *estIa*, encoding for heat-stable enterotoxin of enterotoxigenic *E. coli* (ETEC) (1).

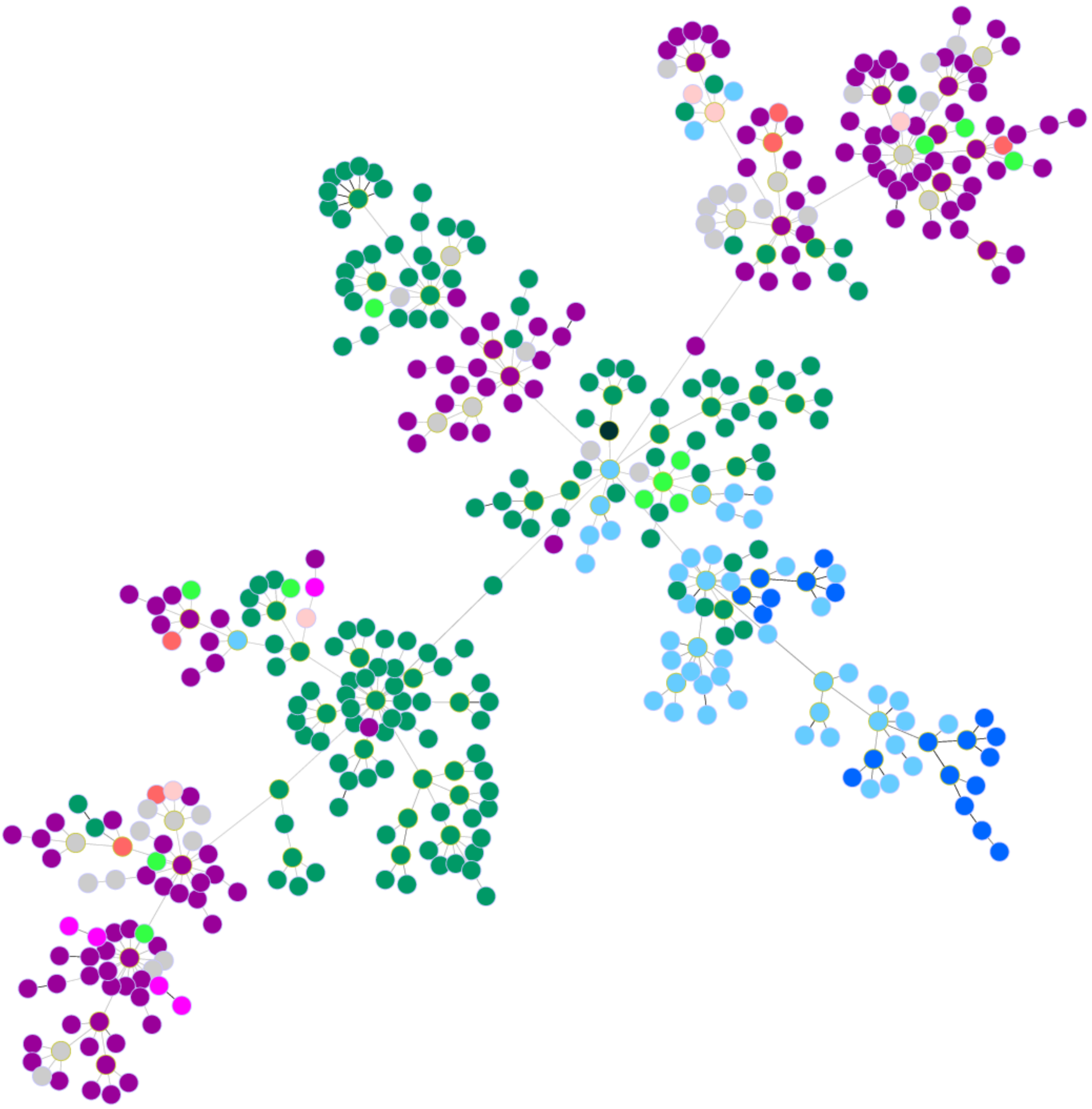

FIG S1 Minimum spanning tree (MST) based on whole-genome multilocus sequence typing (wgMLST) analysis of 32 STEC O157:H7 isolates from this study and 482 globally collected isolates from the INNUENDO database, all representing ST-11 in the 7-loci multilocus sequence typing (MLST). Nodes are colored by geographic regions. Color coding and metadata of the isolates can be found in Data set S1. The isolates of this study (●) grouped with other Finnish isolates (●) in a separate branch from foreign isolates, which predominantly originated from the UK (●) and US (●). The tree was constructed using the goeBURST algorithm in PHYLOViZ (version 2.0) (2).

TABLE S2 WGS and MLST of *C. jejuni* isolates from three dairy farms, collected from February 2014 through May 2015. Data were submitted to the European Nucleotide Archive under project accession no. PRJEB28441. The isolates represent different pulsotypes in pulsed-field gel electrophoresis (PFGE), except the ones denoted with ([b]) after sequence type.

Run accession no.	Alias[a]	Sequence type (ST)	Clonal complex (CC)
ERR2834463	Cj_Farm1_2014-02_C1	1080[b]	-
ERR2834464	Cj_Farm1_2014-03_C1	45	ST-45 CC
ERR2834465	Cj_Farm1_2014-05_C1	1080	-
ERR2834466	Cj_Farm1_2014-12_C1	1080[b]	-
ERR2834467	Cj_Farm2_2014-03_C1	5559	-
ERR2834468	Cj_Farm2_2014-04_C1	677	ST-677 CC
ERR2834469	Cj_Farm2_2014-04_C2	45	ST-45 CC
ERR2834470	Cj_Farm2_2014-05_C1	692	ST-692 CC
ERR2834471	Cj_Farm2_2014-06_C1	11	ST-45 CC
ERR2834472	Cj_Farm2_2014-06_C2	48	ST-48 CC
ERR2834473	Cj_Farm2_2014-07_C1	1701	ST-45 CC
ERR2834474	Cj_Farm2_2014-07_C2	1701	ST-45 CC
ERR2834475	Cj_Farm2_2014-07_C3	1938	-
ERR2834476	Cj_Farm2_2014-08_C1	267	ST-283 CC
ERR2834477	Cj_Farm2_2014-08_C2	45	ST-45 CC
ERR2834478	Cj_Farm2_2014-08_C3	677	ST-677 CC
ERR2834479	Cj_Farm2_2014-09_C1	45	ST-45 CC
ERR2834480	Cj_Farm2_2014-11_C1	7435	-
ERR2834481	Cj_Farm3_2014-02_C1	45	ST-45 CC
ERR2834482	Cj_Farm3_2014-02_C2	925	ST-21 CC
ERR2834483	Cj_Farm3_2014-05_C1	45	ST-45 CC
ERR2834484	Cj_Farm3_2014-05_C2	991	ST-692 CC
ERR2834485	Cj_Farm3_2014-06_C1	4080	-
ERR2834486	Cj_Farm3_2014-06_C2	9407	ST-952 CC
ERR2834487	Cj_Farm3_2014-07_C1	538	ST-45 CC
ERR2834488	Cj_Farm3_2014-07_C2	883[b]	ST-21 CC
ERR2834489	Cj_Farm3_2014-08_C1	538	ST-45 CC
ERR2834490	Cj_Farm3_2014-09_F1	883[b]	ST-21 CC
ERR2834491	Cj_Farm3_2014-10_C1	583	ST-45 CC
ERR2834492	Cj_Farm3_2014-10_C2	925	ST-21 CC
ERR2834493	Cj_Farm3_2014-11_C1	9408	-
ERR2834494	Cj_Farm3_2015-01_C1	883	ST-21 CC
ERR2834495	Cj_Farm3_2015-03_C1	991	ST-692 CC
ERR2834496	Cj_Farm3_2015-03_C2	883	ST-21 CC
ERR2834497	Cj_Farm3_2015-05_C1	883[b]	ST-21 CC
ERR2834498	Cj_Farm3_2015-05_C2	991	ST-692 CC

[a]Aliases indicate the following metadata for the isolates: *C. jejuni*, farm no., isolate collection time (yyyy-mm), source (C=cattle feces, F=milk filter), and colony no.

Farm 1

PFGE	ST	CC	Feb	Mar	Apr	May	Jun	Jul	Aug	Sep	Oct	Dec	Feb
1.01	45	45											
1.02 (=3.07)	45	45											
1.03	1080*	-											
1.04	1080*	-											

Farm 2

PFGE	ST	CC	Mar	Apr	May	Jun	Jul	Aug	Sep	Nov	Jan	Mar	May
2.01	11	45											
2.02	45	45											
2.03	45	45											
2.04	45	45											
2.05	1701	45											
2.06	1701	45											
2.07	48	48											
2.08	267	283											
2.09	677	677											
2.10	677	677											
2.11	692	692											
2.12	1938	-											
2.13 (=3.16)	4080	-											
2.14	5559	-											
2.15	7435	-											

Farm 3

PFGE	ST	CC	Feb	May	Jun	Jul	Aug	Sep	Oct	Nov	Jan	Mar	May
3.04	925	21											
3.05	925	21											
3.01	883*	21											
3.02	883*	21											
3.03	883*	21											
3.06	45	45											
3.07	45	45											
3.08	538	45											
3.09	538	45											
3.10	583	45											
3.11 (=2.08)	267	283											
3.12	991	692											
3.13	991	692											
3.14	991	692											
3.15	9407	952											
3.16	4080	-											
3.17 (=2.14)	5559	-											
3.18	9408	-											

FIG S2 Occurrence of *C. jejuni* PFGE and MLST types on three dairy farms from February 2014 through May 2015. Gray indicates detection of the pulsotype. Dark gray indicates selection of a representative isolate for WGS. Asterix (*) denotes persistent MLST STs on the basis of wgMLST analysis.

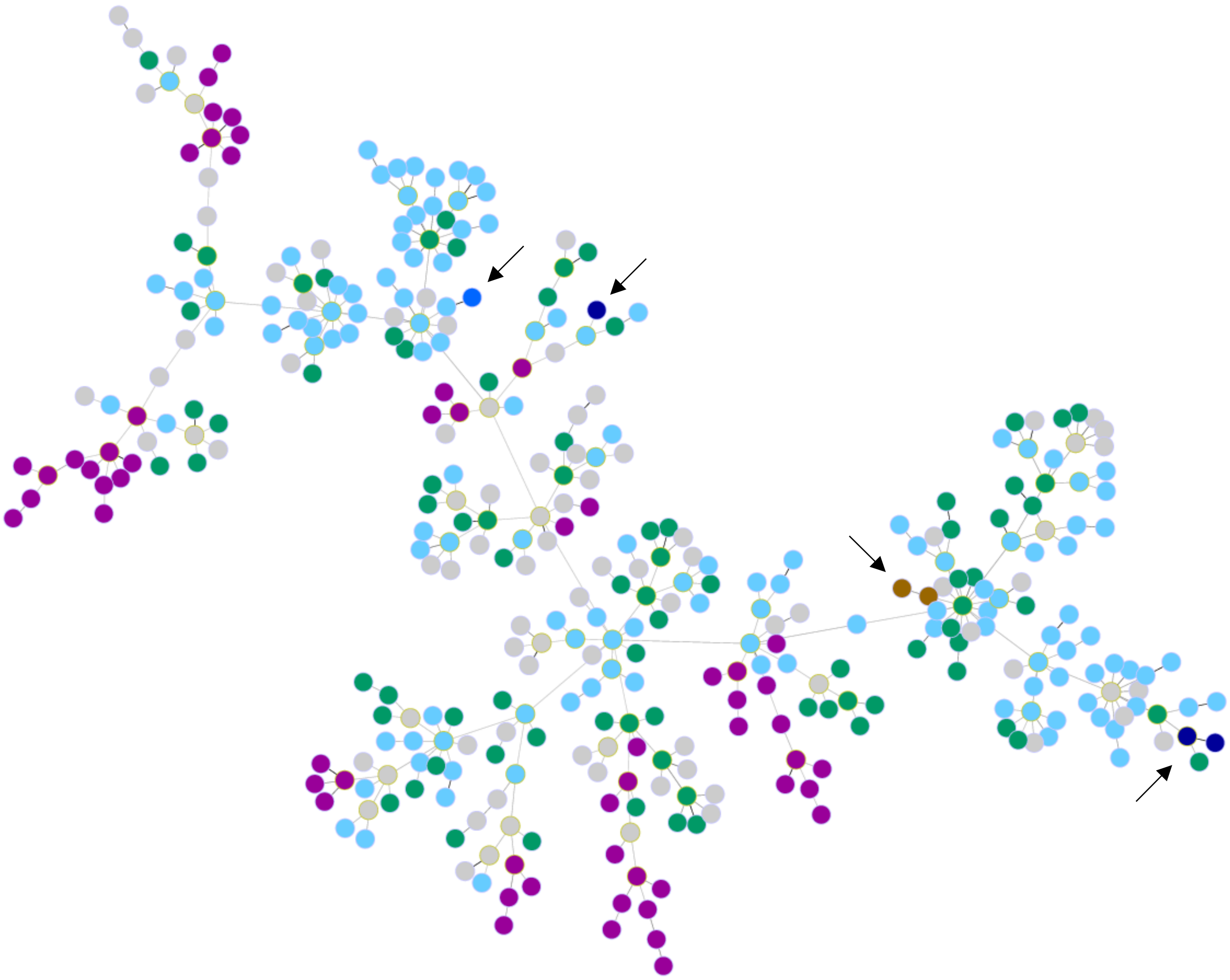

FIG S3 MST based on wgMLST analysis of six ST-45 *C. jejuni* isolates from this study and 436 ST-45 isolates from the INNUENDO database. Nodes are colored by geographic regions. Color coding and metadata of the isolates can be found in Data set S2. Dissimilarity was observed between the isolates collected from farm 2 (●) in spring and fall and between the isolates from three different farms (farm 1: ●, farm 3: ●).

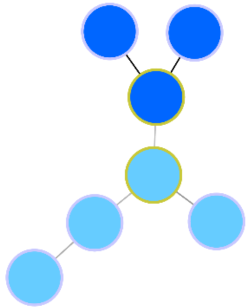

FIG S4 MST based on wgMLST analysis of three ST-1080 *C. jejuni* isolates from this study (farm 1: ●) and four ST-1080 isolates from the INNUENDO database (●), all originating from Finland. Bold links indicate short pairwise distances (PWD 1 or 2). Metadata of the isolates can be found in Data set S3.

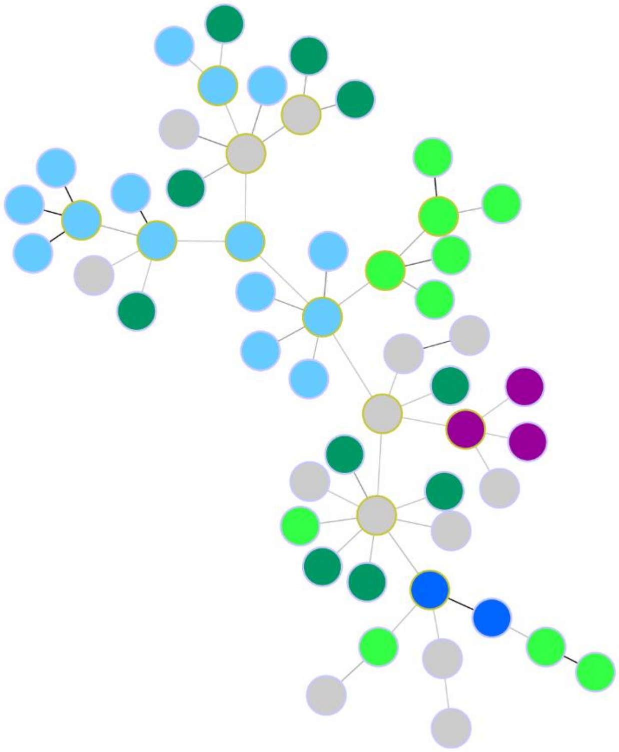

FIG S5 MST based on wgMLST analysis of five ST-883 *C. jejuni* isolates from this study (farm 3: ●) and 66 ST-883 isolates from the INNUENDO database. Color coding and metadata of the isolates can be found in Data set S4. Bold links indicate short distances (PWD 1 or 2). Nodes represent unique allelic profiles. The five isolates of farm 3 represent two unique allelic profiles that differed with PWD of 1.

References

1. Antikainen, J, Tarkka, E, Haukka, K, Siitonen, A, Vaara, M, Kirveskari, J. 2009. New 16-plex PCR method for rapid detection of diarrheagenic *Escherichia coli* directly from stool samples. Eur J Clin Microbiol Infect Dis 28:899–908.

2. Nascimento, M, Sousa, A, Ramirez, M, Francisco, AP, Carrico, JA, Vaz, C. 2017. PHYLOViZ 2.0: providing scalable data integration and visualization for multiple phylogenetic inference methods. Bioinformatics 33:128–129.
